# Cytochalasins from *Xylaria* sp. CFL5, an Endophytic Fungus of *Cephalotaxus fortunei*

**DOI:** 10.1007/s13659-020-00279-5

**Published:** 2020-11-04

**Authors:** Kai-Liang Ma, Shi-Hui Dong, Hang-Ying Li, Wen-Jun Wei, Yong-Qiang Tu, Kun Gao

**Affiliations:** State Key Laboratory of Applied Organic Chemistry, College of Chemistry and Chemical Engineering, Lanzhou University, Lanzhou, 730000 People’s Republic of China

**Keywords:** Cytochalasin, *Xylaria* sp., *Cephalotaxus fortunei*, LAG3/MHC II binding inhibition, LAG3/FGL1 binding inhibition

## Abstract

**Abstract:**

Three previously undescribed cytochalasins, named xylariasins A‒C (**1**‒**3**), together with six known ones (**4**‒**9**) were isolated from *Xylaria* sp. CFL5, an endophytic fungus of *Cephalotaxus fortunei*. The chemical structures of all new compounds were elucidated on the basis of extensive spectroscopic data analyses and electronic circular dichroism calculation, as well as optical rotation calculation. Biological activities of compounds **1**, **4**‒**9** were evaluated, including cytotoxic, LAG3/MHC II binding inhibition and LAG3/FGL1 binding inhibition activities. Compounds **6** and **9** possessed cytotoxicity against AGS cells at 5 μM, with inhibition rates of 94% and 64%, respectively. In addition, all tested isolates, except compound **6**, exhibited obvious inhibitory activity against the interaction of both LAG3/MHC II and LAG3/FGL1. Compounds **1**, **5**, **7**, and **8** inhibited LAG3/MHC II with IC_50_ values ranging from 2.37 to 4.74 μM. Meanwhile, the IC_50_ values of compounds **1**, **7**, and **8** against LAG3/FGL1 were 11.78, 4.39, and 7.45 μM, respectively.

**Graphic Abstract:**

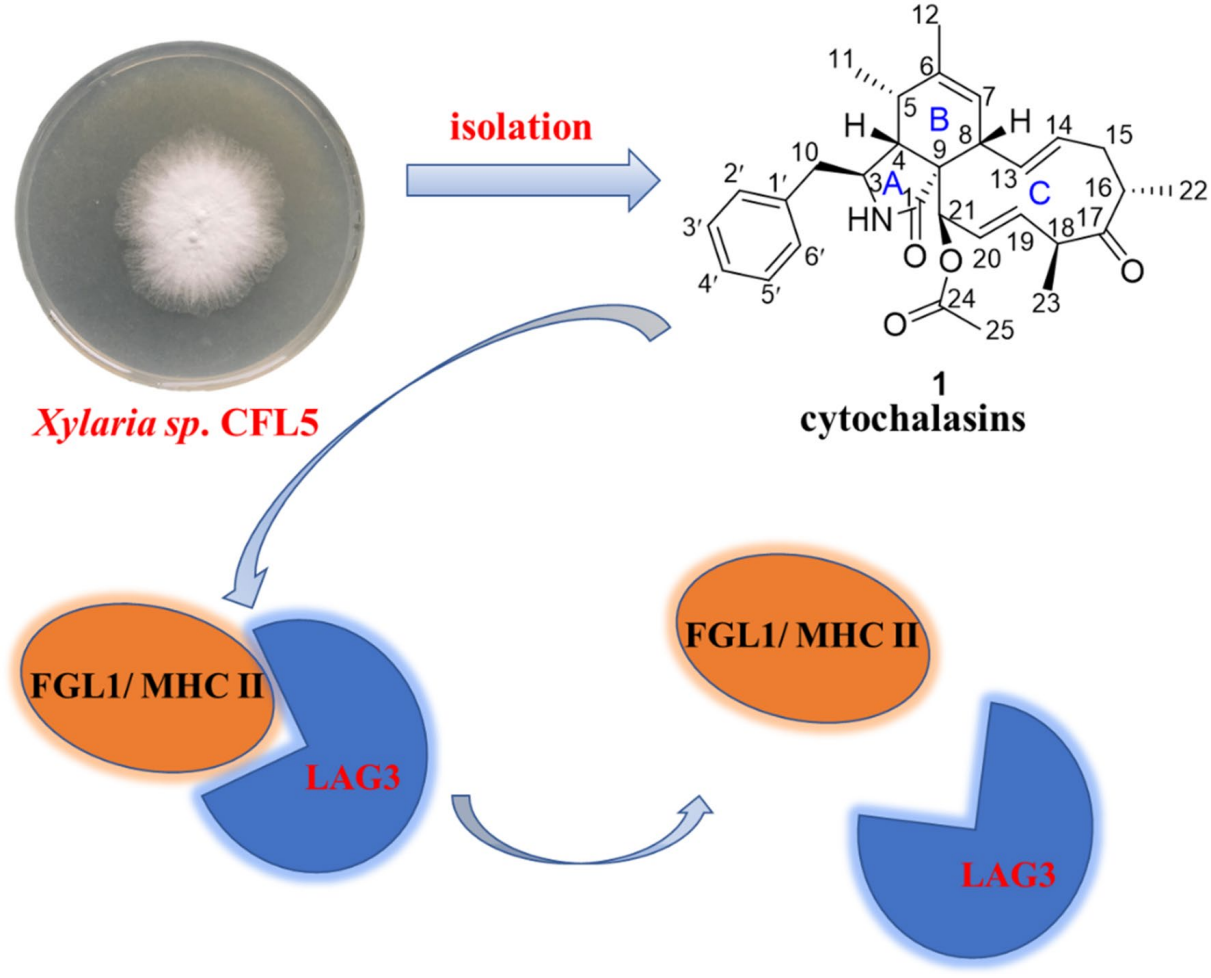

**Electronic supplementary material:**

The online version of this article (10.1007/s13659-020-00279-5) contains supplementary material, which is available to authorized users.

## Introduction

Cytochalasins, a family of fungal metabolites, represented the hybrid formed by a phenylalanine and a polyketide moiety, which possessed unique chemical structures and various biological activities [1, 2]. It was reported that phenylalanine-derived cytochalasans were mostly isolated from the fungi of genera including *Xylaria* [3], *Phomopsis* [4], *Phoma* [5], *Arthrinium* [6], *Aspergillus* [7, 8]. Biological studies have revealed these compounds occupied antibacterial [9], cytotoxic [10], anti-HIV [8], phytotoxic [3], and NO inhibitory activities [11].

*Xylaria* was the largest genus of the family Xylariaceae, and members were widespread on the earth [12]. In the traditional view for this genus, they were saprotrophic fungi usually appeared on deadwood, participated in the decomposition of organic waste, even destructed the growth of plants. Since the fact that members of this genus occur ubiquitously as endophytes of vascular plant has been illuminated, not only *Xylaria*, but the entire Xylariaceae seemed to play an important role in ecology [13]. Previous phytochemical studies on the secondary metabolites of *Xylaria* resulted in the discovery of various compounds with several distinct types of several bioactivities, such as cytochalasin E with phytotoxicity [14] and nigriterpene C possessing anti-inflammatory effects [15], which highlighted the potential of the fungi from this genus as rich sources of natural products with novel chemical structures and valuable bioactivities.

Harringtonin and homobarringtonie from *Cephalotaxus fortunei* had been made numerous biological activity evaluation and formal syntheses works in the past several years [16–18], which also attracted our interests to the secondary metabolites of the endophytic fungi from this plant. A strain of *Xylaria* sp. CFL5 from *C. fortunei* was incubated in rice mediums, a phytochemical investigation resulted in the discovery of nine cytochalasins (**1**‒**9**). After comprehensive structural characterization, including spectroscopic analysis, calculated optical rotation, and calculated electronic circular dichroism, xylariasins A‒C (**1**‒**3**) were identified to possess undescribed structures.

LAG3 (Lymphocyte-activation gene 3) was mainly expressed in activated T and natural killer (NK) cells and was identified as a marker for the activation of CD4^+^ and CD8^+^ T cells [19, 20]. After bound with the cognate ligands, it could be activated to inhibit the proliferation and activation of CD4^+^ and CD8^+^ T cells, enhance the inhibitory activity of regulatory T cells (Treg), and thereby promote the immune escape of tumor cells [21]. MHC II (major histocompatibility complex II) was was believed to be a typical ligand of LAG3 in a long time [22, 23]. However, whether MHC II solely involved in the inhibitory effects of LAG3 or not was controversial. Research workers evidenced that FGL1, a secreted protein from liver cells, was a new LAG3 functional ligand independent of MHC II in 2018 [24]. The discovery revealed a novel immune escape mechanism and provided a guidance for the design of cancer immunotherapy. Collectively, drugs acting on the pathways of either LAG3/MHC II or LAG3/FGL1 could play a role in the cancer therapy. There were only 30 anti-LAG3 monoclonal antibodies in research and few small molecule inhibitors reported [25, 26]. It could provide new lead compounds for cancer immunotherapy and improve the cure rate of cancer through the development of small molecule inhibitors that inhibit the binding of LAG3 with MHC II and the binding of LAG3 with FGL1. For these causes, we evaluated the inhibitory effects of cytochalasins (**1**, **4**‒**9**) from *Xylaria* sp. CFL5 on the interactions of LAG3/MHC II and LAG3/FGL1. The results showed that most isolates, except **6**, exhibited obvious inhibitory activity against the interaction of both LAG3/MHC II and LAG3/FGL1. More specifically, **1**, **5**, **7**, and **8** inhibited LAG3/MHC II with IC_50_ values ranging from 2.37 to 4.74 μM, and the IC_50_ values of **1**, **7**, and **8** against LAG3/FGL1 were 11.78, 4.39, and 7.45 μM, respectively. This was the first report of cytochalasins exhibiting the inhibitory activity against the interaction of LAG3 and MHC II, or LAG3 and FGL1, which underscored the potential of cytochalasin derivatives as anticancer immunosuppressants.

## Results and Discussion

### Isolation and Structure Elucidation

**Fig. 1:**
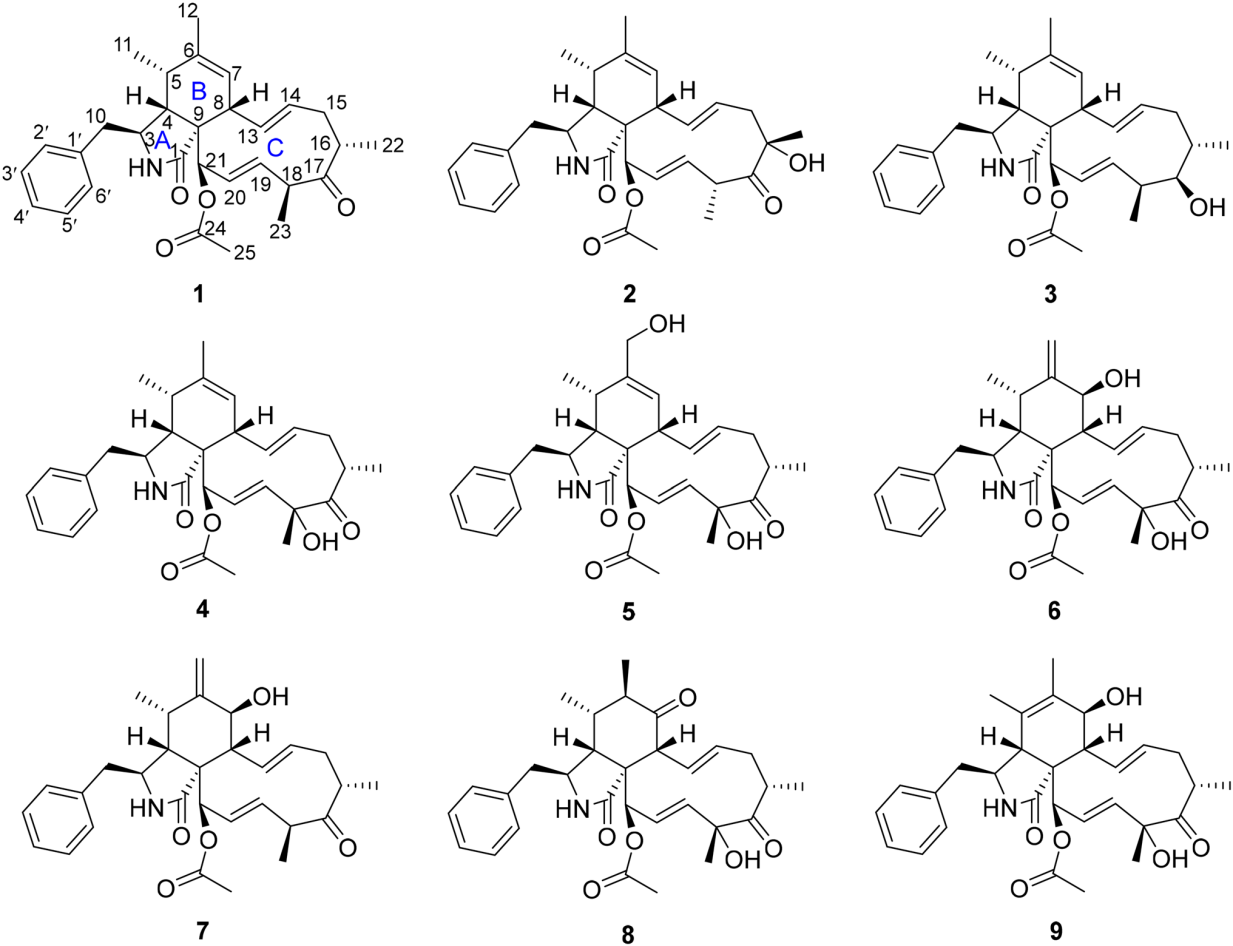
Structures of compounds **1**–**9**

The strain of *Xylaria* sp. CFL5 was inoculated on rice mediums and cultured at 28 °C for 20 days. Utilizing macroporous resin HP-20, silica gel column chromatography, and semi-preparative HPLC, the investigation of the EtOAc extract of the mediums provided three new (**1**‒**3**) and six published (**4**‒**9**) cytochalasins (Fig. 1). The structures and stereochemistry of these isolates were elucidated on the basis of spectroscopic analysis, optical rotation, and circular dichroism (CD) analysis and comparison with the data in the literature.

**Fig. 2:**
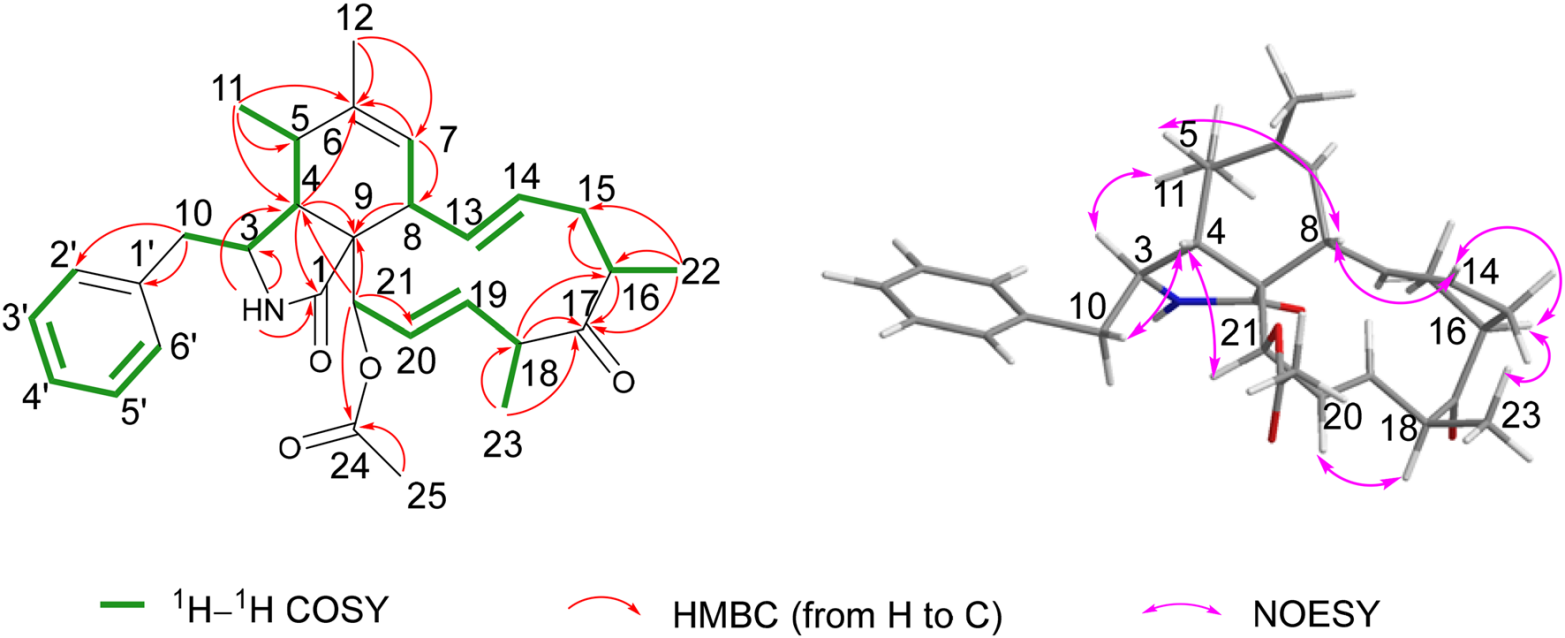
Key ^1^H–^1^H COSY, HMBC, and NOESY correlations of compound **1**

Compound **1** was isolated as a white powder. Its molecular formula was determined as C_30_H_37_NO_4_ according to an [M + H]^+^ ion peak at *m/z* 476.2793 (calcd 476.2795) in the HRESIMS spectrum, showing 13 indices of hydrogen deficiency (IOHDs). The characteristic absorptions at 1743 and 1703 cm^−1^ for carbonyl groups, as well as the bands at 3324 cm^−1^ for amino groups in its IR spectrum were observed. The ^1^H, ^13^C NMR, and HSQC spectra for compound **1** revealed three methyl doublets at (*δ*_H_ 1.10, *J* = 7.6 Hz; *δ*_C_ 14.2), (*δ*_H_ 1.15, *J* = 6.8 Hz; *δ*_C_ 19.1), and (*δ*_H_ 1.22, *J* = 7.2 Hz; *δ*_C_ 16.3), two methyl singlets at (*δ*_H_ 1.69; *δ*_C_ 19.9) and (*δ*_H_ 2.23; *δ*_C_ 20.9). The resonance signals in the ^1^H NMR at *δ*_H_ 7.12 (d, *J* = 6.8 Hz, 2H), 7.30 (t, *J* = 6.8 Hz, 2H), and 7.24 (m, 1H) disclosed that there was a monosubstituted phenyl moiety in the molecule, which was evidenced by the ^13^C NMR signals at *δ*_C_ 129.2 (2C), 129.0 (2C), 127.2 (1C), and 137.6 (1C). In addition, three pairs of double bands were also determined based on the signals at (*δ*_H_ 5.80, dd, *J* = 16.0 Hz, *J* = 10.0 Hz; *δ*_C_ 132.1), (*δ*_H_ 5.08, ddd, *J* = 15.2 Hz, *J* = 10.8 Hz, *J* = 4.4 Hz; *δ*_C_ 132.4), (*δ*_H_ 4.75, ddd, *J* = 16.0 Hz, *J* = 7.6 Hz, *J* = 2.4 Hz; *δ*_C_ 124.5), (*δ*_H_ 6.04, ddd, *J* = 16.0 Hz, *J* = 2.8 Hz, *J* = 1.2 Hz; *δ*_C_ 132.6), (*δ*_H_ 5.31, s; *δ*_C_ 127.7), and (*δ*_C_ 138.1) in the ^1^H and ^13^C NMR data. In conjunction with the 1D and 2D NMR data, the remaining carbon signals were designated two methylenes, six methines, an oxygenated tertiary carbon, a sp^3^ quarternary carbon, and three carbonyl groups. Unambiguously, these evidences exhibited that compound **1** was a cytochalasin derivative [4, 27]. The polyketide moiety of **1** was constructed on the basis of the ^1^H–^1^H COSY couplings of H-21/H-20/H-19/H-18/H-23 and H-7/H-8/H-13/H-14/H-15/H-16/H-22 and HMBC cross-peaks from H_3_-11 to C-5 and C-6, from H_3_-12 to C-6 and C-7, from H-7 to C-6 and C-8, from H-8 to C-9, from H-21 to C-1, C-9, and C-20, from H_3_-22 to C-15, C-16, and C-17, from H-16 to C-15 and C-17, from H_3_-23 to C-18 and C-17, and from H-18 to C-17 and C-16 (Fig. 2). The moiety of phenylalanine was established by the ^1^H–^1^H COSY couplings of H-2′/H-3′/H-4′/H-5′/H-6′ and HMBC cross-peaks from H-10 to C-1′, C-2′, C-3, and C-4, from H-4 to C-1 and C-9, and from H-21 to C-4 and C-9, from NH to C-1, C-3, and C-4, and from H-3 to C-1′ (Fig. 2). Ultimately, the linkage between these two moieties was deduced from the ^1^H–^1^H COSY correlations of H-11/H-5/H-4/H-3/H-10 and HMBC cross-peaks from NH to C-1 and C-9, from H-11 to C-4, and from H-4 to C-5 and C-6, as depicted in Figs. 1 and 2. The chemical shift of C-21 (*δ*_H_ 5.53, s; *δ*_C_ 77.2) and the HMBC cross-peaks of H-21 to C-24 and H_3_-25 to C-24 revealed that the acetyl group was connected to C-21. Herein, the planar structure of compound **1** was determined as depicted in Fig. 1.

**Fig. 3:**
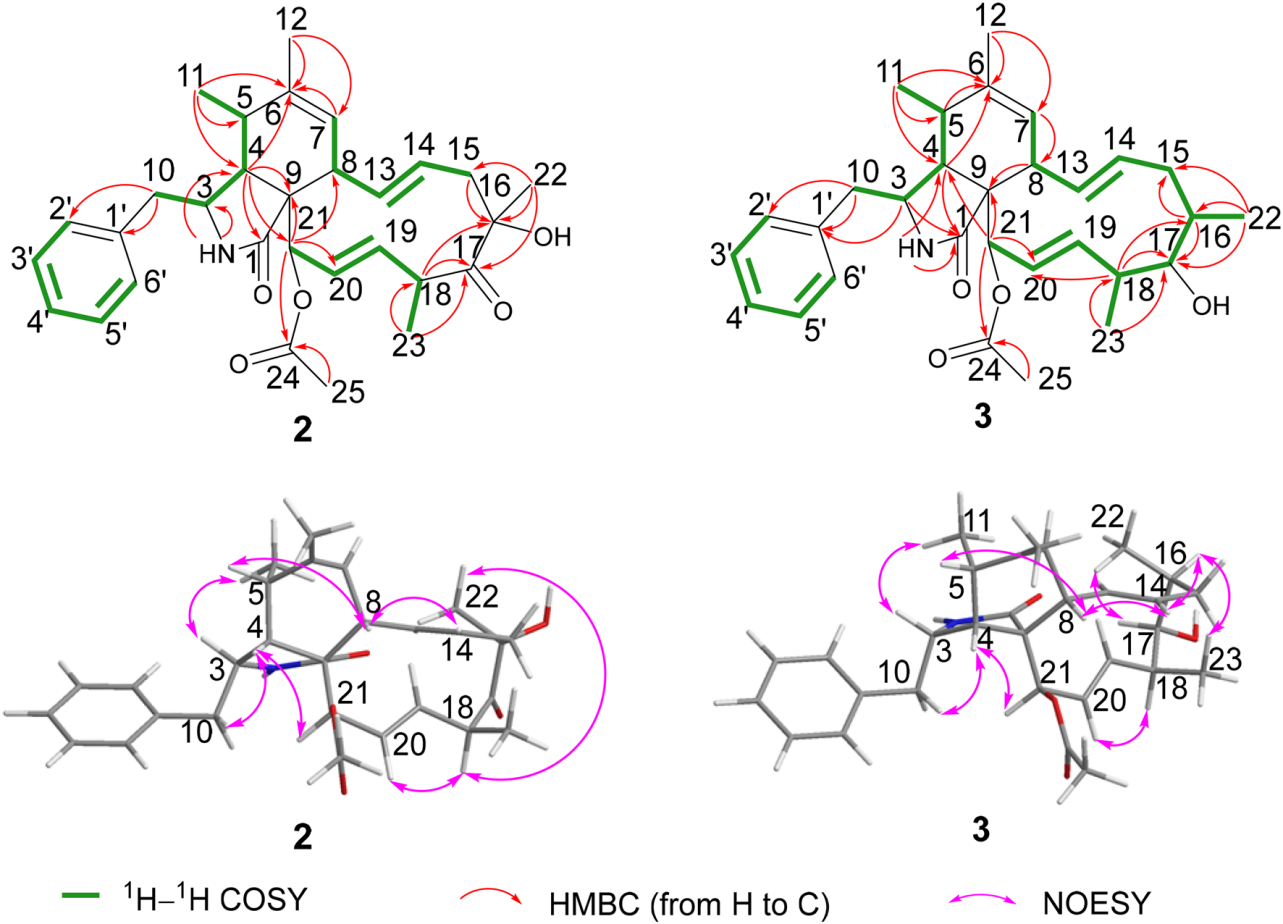
Key ^1^H–^1^H COSY, HMBC, and NOESY correlations of compounds **2** and **3**

The relative configuration of compound **1** was determined by the NOESY experiment. The NOESY correlations of H-5/H-8, H_3_-11/H-3, H-4/H-10a suggested that H-8, H-4, and H_2_-10 were cofical and assigned as *β* orientation. Accordingly, H_3_-11 and H-3 were *α* oriented. The correlations of H-16 with H_3_-23, H-8 with H-14, H-14 with H-16, and H-4 with H-21 in the NOESY spectrum revealed that H_3_-22 and H-21 were *α* oriented, the configuration of C-9 was S*, H_3_-23 was *β* oriented, and the Δ^13^ double bond was *E*-configuration [27, 28]. The Δ^19^ double bond was *E*- configuration on the basis of the NOESY correlation of H-18 and H-20. Subsequently, these data confirmed the relative configuration of **1**, which offered two possible stereo-structure candidates assembled as **1a** (3*S*,4*R*,5*S*,8*S*,9*S*,16*S*,18*S*, 21*R*) and **1b** (3*R*,4*S*,5*R*,8*R*,9*R*,16*R*,18*R*,21*S*). The ECD spectra of both **1a** and **1b** were calculated. The ECD values for **1a** showed negative (212 nm and 308 nm) and positive (245 nm) cotton effects, the shape of which matched with that of the experimental curve (Fig. 3). Thus, the absolute configuration of compound **1** was determined as 3*S*,4*R*,5*S*,8*S*,9*S*,16*S*,18*S*,21*R*, and the name of xylariasin A was proposed for **1**.

**Fig. 4:**
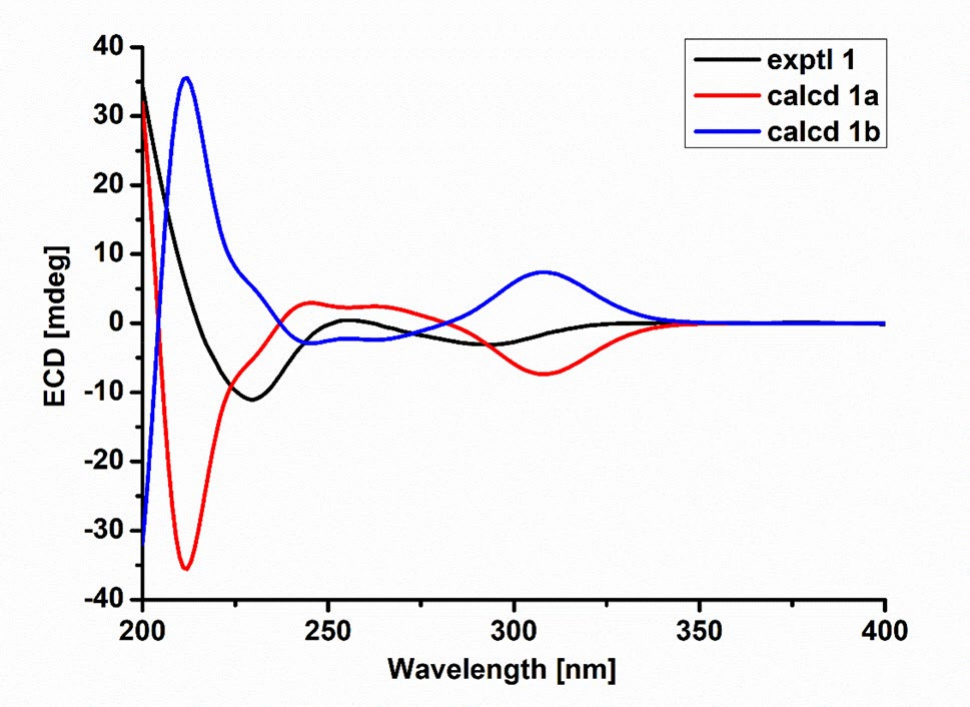
ECD spectra of compound **1** (solvent: CH_3_OH)

**Fig. 5:**
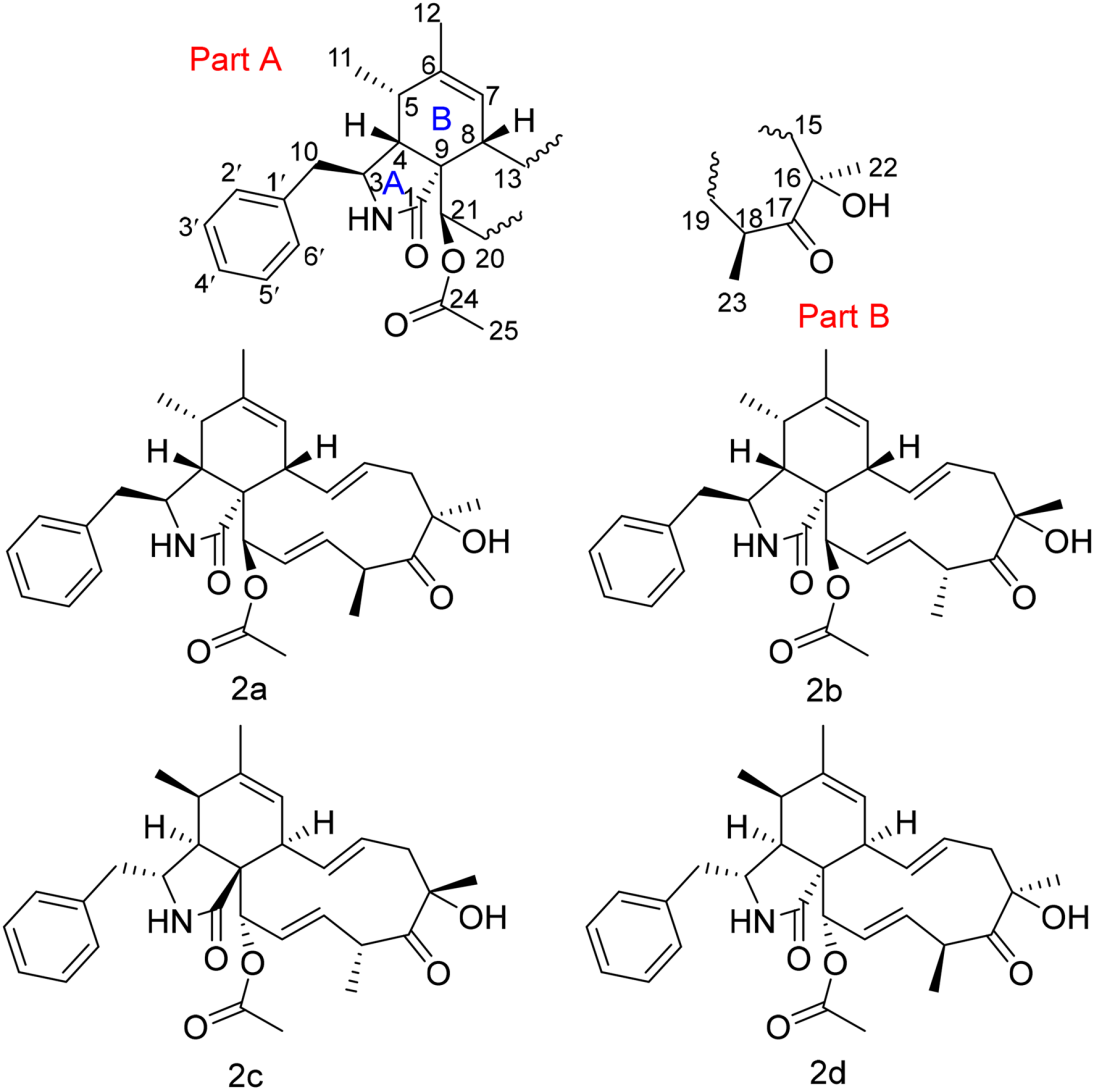
The probable absolute configuration for compound **2**

Compound **2**, a coreless oil, which was evidenced to possess a molecular formula of C_30_H_37_NO_5_ from its ( +)-HRESIMS ion at *m/z* 492.2741 ([M + H]^+^, calcd for C_30_H_38_NO_5_ 492.2744) and ^13^C NMR data, indicating the presence of 13 IOHDs. Absorption bands at 1740 and 1692 cm^−1^ (carbonyl groups), and 3456 and 3302 cm^−1^ (amino/hydroxyl groups) in the IR spectra were designated. The characteristic resonances included signals for a monosubstituted phenyl moiety at *δ*_H_ 7.12 (d, *J* = 6.8 Hz, 2H), 7.30 (t, *J* = 6.8 Hz, 2H), and 7.24 (m) and five methyl groups at *δ*_H_ 1.15 (d, *J* = 7.2 Hz), 1.19 (d, *J* = 6.8 Hz), 1.71 (s), 1.42 (s), and 2.21 (s), and olefinic and oxygenated protons at *δ*_H_ 5.25 (s), 6.18 (s), 4.94 (m), 4.98 (m), 6.21 (s), 5.49 (s), and 6.05 (s) in the ^1^H NMR spectrum. These data are reminiscent of compound **1**, suggesting that compound **2** was a similar cytochalasin derivative. The main difference between these two compounds in the ^1^H NMR spectra was a methyl doublet in **1** became a methyl singlet in **2**. Accordingly, the most obvious difference in their ^13^C NMR spectra was the absence of a methine carbon in **1** and the presence of an oxygenated tertiary carbon at *δ*_C_ 81.9 in **2**. These data supported a hypothesis that there was a hydroxyl group connected to C-16, which was confirmed by the HMBC cross-peaks from H-15 to C-16, from H_3_-22 to C-15, C-16, and C-17, and from H-18 to C-16 and C-17. Thus, the planar structure of compound **2** was demonstrated as Fig. 4. The NOESY correlations of H-5/H-8, H-3/H_3_-11, H-4/H-10, and H-4/H-21 revealed these protons possessed the same relative configurations with those of compound **1**, which was demonstrated as part A in Fig. 5. The NOESY correlations of H-8/H-14 and H-20/H-18 revealed the Δ^13^ and Δ^19^ double bonds were *E*-configuration. The correlation between H_3_-22 and H-18 in the NOESY spectrum revealed that H_3_-22 and H_3_-23 were located on the opposite side, described as part B in Fig. 5. However, H-16 in **1** was replaced by a hydroxyl group in compound **2**, resulting in the absence of NOESY resonance signal of H-14/H-16. The relative configurations of part A and part B cannot be coordinated based on the NOESY correlations, which gave two possibilities depicted as **2a** and **2b** in Fig. 5. Accordingly, four possible enantiomers **2a**‒**2d** (Fig. 5) were inferred to comprise the absolute configuration of **2**.

**Fig. 6:**
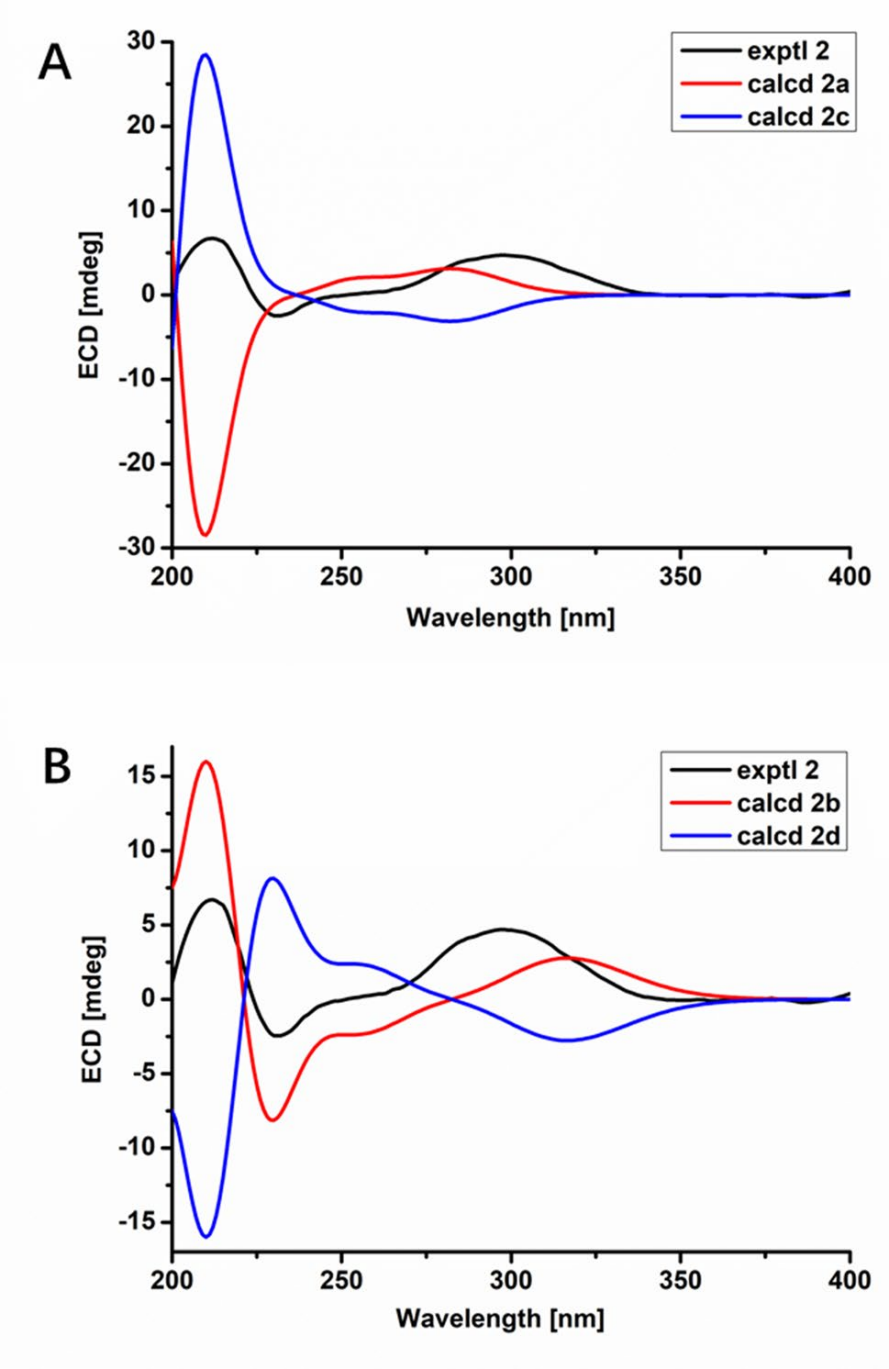
ECD spectra of compound **2** (solvent: CH_3_OH)

The ECD spectra of **2a**‒**2d** were calculated through the density functional theory (DFT) optimization at the level of b3lyp/6-31 g(d,p). The calculated ECD values of (3*S*,4*R*,5*S*,8*S*,9*S*,16*S*,18*R*,21*R*)-**2** (**2b**) exhibited good agreement with the experimental ECD values of **2** (Fig. 6), indicating **2** was 3*S*,4*R*,5*S*,8*S*,9*S*,16*S*,18*R*,21*R*. Accordingly, the calculated optical rotation for **2b** was + 9.55, which was closer to the experimental data of **2** ($[\alpha ]_{{\text{D}}}^{19}$ =  + 13.559), supporting the absolute configuration of **2**. Compound **2** was determined as depicted in Fig. 1 and defined as xylariasin B. The hydroxyl substituent at C-16 in cytochalasins was rare, and compound **2** was the first example, which indicated that there may be an oxidase responsible for hydroxylation of C-16 in the biosynthesis pathway.

**Fig. 7:**
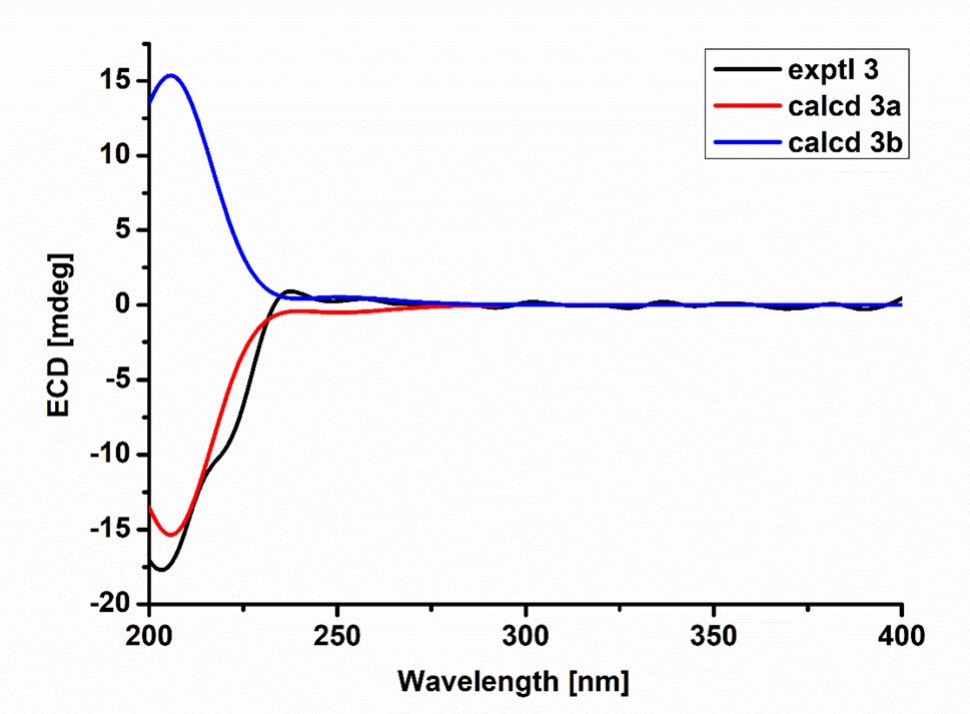
ECD spectra of compound **3** (solvent: CH_3_OH)

Compound **3**, a white powder, had a molecular formula of C_30_H_39_NO_4_, requiring 12 IOHDs, which was established by the ^13^C NMR data and a sodium adduct ion peak at *m*/*z* 500.2768 ([M + Na]^+^, calcd 500.2771) in the HRESIMS spectrum. Inspection of the ^1^H NMR spectrum with that of compound **1** suggested that they were analogues. The major difference was that there was an additional oxygenated methine proton at *δ*_H_ 3.79 in compound **3**. Correspondingly, an oxygenated methine carbon replacing the carbonyl group was observed in the ^13^C NMR spectrum of compound **3**. These data gave a deduction that the carbonyl group at C-17 was changed to a hydroxyl group, in agreement with the fact that compound **3** possessed a less IOHD than compound **1.** This assumption was subsequently supported by the key ^1^H–^1^H COSY correlations and the key HMBC correlations as depicted in Fig. 3. The NOESY correlation of H-17 with H_3_-22 suggested that H-17 was *α*-oriented, in conjunction with the same NOESY correlations of compound 3 as those of compound 1, which gave two possibilities for the absolute configuration of compound **3**, assigned as **3a** (3*S*,4*R*,5*S*,8*S*,9*S*,16*S*,17*R*,18*S*,21*R*) and **3b** (3*R*,4*S*,5*R*,8*R*, 9*R*,16*R*,17*R*,18*R*,21*S*). The ECD curve of **3a** was consistent with that of experimental curve (Fig. 7), evidencing the absolute configuration of **3** was 3*S*, 4*R*, 5*S*, 8*S*, 9*S*, 16*S*, 17*R*, 18*S*, 21*R*. Further experimental specific rotation for compound **3** was − 20.883, approximating that of compound 1 ($[\alpha ]_{{\text{D}}}^{19}$ =  − 18.853), which unambiguously certified the absolute configuration of **3**. Thus, the structure of compound **3** was demonstrated in Fig. 1 and named xylariasin C.

By comparison with the published ^1^H and ^13^C NMR data, the six known cytochalasins were identified as zygosporin G (**4**) [29],12-hydroxyl zygosporin G (**5**) [30], cytochalasin D (**6**) [30, 31], zygosporin E (**7**) [30], compound 10 (**8**) [32], and cytochalasin C (**9**) [33].

Expect xylarasins B (**2**) and C (**3**), the cytotoxicity, LAG3/MHC II inhibition activity, and LAG3/FGL1 inhibition activity of all isolates were tested.

### Cytotoxic Activity

Cytochalasin D (**6**) and cytochalasin C (**9**) exhibited strong cytotoxic activity against AGS cells. At a concentration of 5 μM, cytochalasin D (**6**) and cytochalasin C (**9**) possessed inhibition rates of 94% and 64%, respectively.

### LAG3/MHC II Binding Inhibition Activity

**Fig. 8:**
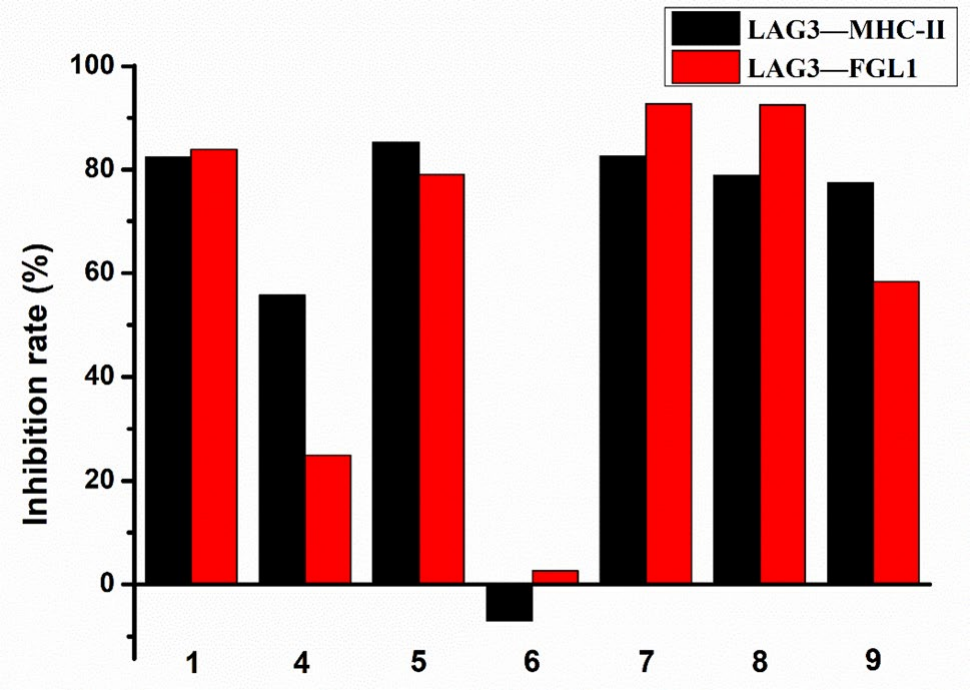
The inhibition rates of tested compounds (**1**, **4**‒**9**) against LAG3/MHC II and LAG3/FGL1 at a concentration of 50 μM

To explore anticancer immunosuppression activity of cytochalsins from *Xylaria* sp. CFL5, the effects of all isolates except xylariasins B (**2**) and C (**3**) against the binding between LAG3 and MHC II were investigated. As shown in Table 3 and Fig. 8, cytochalasin D (**6**) regrettably did not reveal obvious inhibition effects on the interaction between these two proteins, while zygosporin G (**4**) possessed an inhibition rate of 55.72% at the concentration of 50 μM. It was found that all the other tested compounds (**1**, **5**, **7**‒**9**) inhibited the binding of LAG3 protein and MHC II protein, exhibiting inhibition rates approximately 80%. Compounds **1**, **5**, **7**, **8** possessed IC_50_ values ranging from 2.37 to 4.74 μM, as demonstrated in Table 4.

### LAG3/FGL1 Binding Inhibition Activity

LAG3/FGL1 was a novel immunosuppression pathway independent of LAG3/MHC II. The inhibitors influencing the binding of LAG3 and FGL1 could also prevent tumor immune escape, which could make the tumor cells recognized and killed by T cells. Exploiting the inhibitors acting on the LAG3/FGL1 pathway could provide a possibility to the cancer immunotherapy. The inhibition activity of compounds **1**, **4**‒**9** against the interaction of LAG3/FGL1 were evaluated in vitro. The result was summarized in Fig. 8 and Tables 3 and 4. At the concentration of 50 μM, compounds **4** and **9** exhibited moderate inhibition activity on the LAG3/FGL1, with inhibition rates of 24.90% and 58.36%, respectively. Compounds **1**, **5**, **7**, and **8** possessed inhibition rates ranging from 79.11% to 92.70% at the same concentration. The IC_50_ values of compounds **1**, **7**, **8** against the binding between LAG3 and FGL1 were 11.78, 4.39, and 7.45 μM, respectively.

In summary, nine cytochalasins including three undescribed ones (**1**‒**3**) were isolated from *Xylaria* sp. CFL5, an endophytic fungus of *Cephalotaxus fortunei*. Interestingly, all tested isolates possessed biological activities, including cytotoxicity, LAG3/FGL1 binding inhibition activity, and LAG3/FGL1 binding inhibition activity. Cytochalasin D (**6**) and cytochalasin C (**9**) possessed cytotoxicity against AGS cells at 5 μM, with inhibition rates of 94% and 64%, respectively. Except cytochalasin D (**6**), all tested compounds simultaneously showed inhibition effects on the interaction of LAG3 and MHC II, or LAG3 and FGL1 (Fig. 8). Compound **1**, **5**, **7**, and **8** inhibited LAG3/MHC II with IC_50_ values ranging from 2.37 to 4.74 μM. Meanwhile, the IC_50_ values of compounds **1**, **7**, and **8** against LAG3/FGL1 were 11.78, 4.39, and 7.45 μM, respectively. Drugs acting on either LAG3/MHC II or LAG3/FGL1 pathway can play a part in preventing tumor immune escape. However, there were few small molecule inhibitors acting on LAG3/MHC II or LAG3/FGL1, and drugs targeting LAG3 are basically antibodies. Our research results supplemented the gap of small inhibitors on these two pathways, and it was the first report that cytochalasin derivatives can break the interactions of both LAG3/MHC II and LAG3/FGL1. These data provided cytochalasin derivatives with more possibilities of biological activities, highlighting the application potential in cancer immunotherapy.

## Experimental

### General Experimental Procedures

The optical rotations were measured using a PerkinElmer model 341 polarimeter. A Bruker Tensor spectrometer was used for the measurement of the IR spectra. A Shimadzu UV-260 spectrophotometer was used to measure the UV spectra. ^1^H, ^13^C, and 2D NMR spectra were recorded on a Bruker AVANCE III-400, a Bruker AVANCE NEO 600, or a Varian Mercury-600BB spectrometer. The HRESIMS data were acquired utilizing a Bruker Daltonics APEX II spectrometer. The ECD curves were obtained on an Olis DSM-1000 spectrometer. The compounds were purified by a macroporous resin HP-20 and a semipreparative HPLC with a reversed-phase C_18_ (150 × 10 mm, 10 μm) column. Silica gel (200–300 mesh) for column chromatography and Silica gel GF254 (10–40 mm) for TLC were purchased from the Qingdao Marine Chemical Factory, Qingdao, China.

### Fungal Material

The fungus was obtained from the leaves of *Cephalotaxus fortunei* Hook. The plant sample was collected from Feng County, Shaanxi Province, China in August, 2017, which was identified by the professor Huyuan Feng (School of Life Science, Lanzhou University). On the basis of the ITS sequence analysis, the fungus was identified as *Xylaria* sp. (Genbank: KU645984.1). The fungus was inoculated on a PDA plate to obtain mycelium. Then its mycelium was picked into an Erlenmeyer flasks (100 mL) with 50 mL of YMG medium, cultured at 28 °C, 140 rpm for 5 days. 2 mL of mediums were transferred into three Erlenmeyer flasks (500 mL) with 200 mL of YMG medium. After cultivation at the same condition for 7 days, the seed culture was given. Sequently, 10 mL of seed culture was carried out in 50 rice mediums (80 g rice, 4 g sucrose, 120 mL distilled water) and incubated at 28 °C for 20 days.

### Extraction, Isolation, and Purification Process

The solid mediums were extracted with EtOAc for three times. After removing the solvent under reduced pressure, a crude extract (53.9 g) was given. The crude extract was submitted to a macroporous resin HP-20 chromatographic column and eluted with a gradient elution (30%, 50%, 80%, 95% EtOH) to yield four fractions (Fr. 1–4). Fr. 3 (17.2 g) was purified using a silica gel column with a gradient elution (petroleum ether–ethyl acetate, 10:1 to 1:2) as eluent. Seven fractions (Fr. 3A–3G) were given after combination and concentration with TLC analysis. Fr. 3B (400 mg) was purification with a reversed-phase semipreparative HPLC (CH_3_CN/H_2_O, 7/3, v/v) to give compounds **1** (4.7 mg, t_R_ = 30 min) and **2** (2.6 mg, t_R_ = 16 min). Compound **4** (20 mg, t_R_ = 38 min) was obtained from Fr. 3C (560 mg) with a reversed-phase semipreparative HPLC (60% CH_3_CN). The purification of Fr. 3D (1.4 g) with a reversed-phase semipreparative HPLC (85% CH_3_OH) yielded compound **3** (2.2 mg, t_R_ = 20 min). Fr. 3E (3.7 g) was isolated with a reversed-phase semipreparative HPLC (CH_3_CN/H_2_O, 115/85, v/v) to obtain compounds **8** (3.2 mg, t_R_ = 50 min) and **9** (2.8 mg, t_R_ = 60 min). Using (MeCN/H_2_O = 101:99, v/v) as the eluent, compounds **5** (80.2 mg, t_R_ = 18 min), **6** (7.7 mg, t_R_ = 22 min), and **7** (2.4 mg, t_R_ = 32 min) were purified from Fr. 3F (4.7 g) by a reversed-phase semipreparative HPLC.

Xylariasin A (**1**): white powder; $[\alpha ]_{{\text{D}}}^{19}$ =  − 18.853 (MeOH, *c* 1.273); IR (KBr) *ν*_max_ 3324, 2963, 2926, 1743, 1703, 1604, 1456, 1227, 1098, 1037, 737, 702 cm^−1^; ECD (MeOH) *λ*_max_ (Δ*ε*), 229 (− 10.57), 256 (+ 0.43), 294 (− 3.10) nm; HRESIMS [M + H]^+^ *m/z* 476.2793 (calcd for C_30_H_38_NO_4_ 476.2795); ^1^H and ^13^C NMR data, found in Tables 1 and 2.

**Table 1:** ^1^H NMR Spectroscopic Data (in CDCl_3_, *J* in Hz) for Compounds **1**, **2**, and **3**

Position	**1**^a^	**2**^a^	**3**^b^
3	3.16, dd (8.4, 4.0)	3.13, m	3.17, m
4	2.13, t (4.4)	2.14, t (4.0)	2.16, t (4.2)
5	2.42, br s	2.41, br s	2.45, br s
7	5.31, s	5.25, s	5.37, s
8	3.12, m	3.08, d (8.8)	3.18, m
10	a 2.87, dd (13.6, 4.4)	a 2.92, dd (13.6, 3.6)	a 2.89, dd (13.8, 4.2)
	b 2.57, dd (13.6, 9.6)	b 2.53, dd (13.6, 10.0)	b 2.59, dd (13.2, 9.6)
11	1.10, d (7.6)	1.15, d (7.2)	1.13, d (7.2)
12	1.69, s	1.71, s	1.71, s
13	5.80, dd (16.0, 10.0)	6.18, s	5.85, dd (15.6, 10.2)
14	5.08, ddd (15.2, 10.8, 4.4)	4.94, m	5.09, ddd (15.6, 10.8, 4.8)
15	a 2.29, m	a 2.69, m	a 1.92, dd (12.6, 4.8)
	b 2.00, dt (12.8, 6.0)	b 2.61, m	b 1.87, t (12.0)
16	2.64, ddd (9.6, 6.8, 2.4)		1.57, m
17			3.79, s
18	3.25, t (7.2)	3.72, m	2.50, m
19	4.75, ddd (16.0, 7.6, 2.4)	4.98, m	4.86, ddd (15.0, 7.2, 1.8)
20	6.04, ddd (16.0, 2.8, 1.2)	6.21, s	6.00, d (16.2)
21	5.53, s	5.49, s	5.60, s
22	1.15, d (6.8)	1.42, s	0.99, d (7.2)
23	1.22, d (7.2)	1.19, d (6.8)	0.98, d (6.6)
25	2.23, s	2.21, s	2.23, s
2′/6′	7.12, d (6.8)	7.12, d (6.8)	7.14, d (7.8)
3′/5′	7.30, t (6.8)	7.31, t (7.6)	7.32, t (7.2)
4′	7.24, m	7.26, m	7.25, m
NH	5.89, s	6.05, s	5.43, s

^a^Recorded in 400 MHz, TMS, *δ* in ppm

^b^Recorded in 600 MHz, TMS, *δ* in ppm

**Table 2:** ^13^C NMR Spectroscopic Data (Recorded in 150 MHz, in CDCl_3_) for compounds **1**, **2**, and **3**

Position	1	2	3
1	175.3, C	176.1, C	175.3, C
3	55.8, CH	56.0, CH	55.8, CH
4	53.8, CH	53.5, CH	54.5, CH
5	35.3, CH	35.2, CH	35.4, CH
6	138.1, C	138.5, C	137.8, C
7	127.7, CH	127.2, CH	128.1, CH
8	43.0, CH	42.1, CH	43.3, CH
9	57.4, C	55.6, C	57.0, C
10	45.9, CH_2_	45.8, CH_2_	46.1, CH_2_
11	14.2, CH_3_	14.7, CH_3_	14.1, CH_3_
12	19.9, CH_3_	20.1, CH_3_	20.0, CH_3_
13	132.1, CH	135.3, CH	130.1, CH
14	132.4, CH	129.0, CH	134.0, CH
15	37.6, CH_2_	42.7, CH_2_	40.8, CH_2_
16	44.0, CH	81.9, C	32.8, CH
17	211.8, C	216.3, C	78.5, CH
18	50.2, CH	46.3, CH	43.0, CH
19	124.5, CH	127.2, CH	131.2, CH
20	132.6, CH	131.4, CH	129.6, CH
21	77.2, CH	76.7, CH	77.5, CH
22	19.1, CH_3_	25.5, CH_3_	17.1, CH_3_
23	16.3, CH_3_	18.7, CH_3_	12.3, CH_3_
24	170.0, C	170.0, C	170.2, C
25	20.9, CH_3_	20.1, CH_3_	21.0, CH_3_
1′	137.6, C	137.5, C	137.9, C
2′/6′	129.2, CH	129.0, CH	129.1, CH
3′/5′	129.0, CH	129.0, CH	129.1, CH
4′	127.2, CH	127.4, CH	127.2, CH

Xylariasin B (**2**): colorless oil; $[\alpha ]_{{\text{D}}}^{19}$ =  + 13.559 (MeOH, *c* 0.885); IR (KBr) *ν*_max_ 3456, 3302, 2969, 2933, 1740, 1692, 1454, 1373, 1229, 738, 702 cm^−1^; ECD (MeOH) *λ*_max_ (Δ*ε*), 212 (+ 6.70), 231 (− 2.44), 243 (− 0.50), 266 (+ 0.70), 298 (+ 4.70) nm; HRESIMS [M + H]^+^ *m/z* 492.2741 (calcd for C_30_H_38_NO_5_ 492.2744); ^1^H and ^13^C NMR data, found in Tables 1 and 2.

Xylariasin C (**3**): white powder; $[\alpha ]_{{\text{D}}}^{19}$ =  − 20.883 (MeOH, *c* 0.336); IR (KBr) *ν*_max_ 3369, 2925, 1773, 1719, 1686, 1262, 1232, 1107, 1074, 1027, 740, 702 cm^−1^; ECD (MeOH) *λ*_max_ (Δ*ε*), 203 (− 17.70), 222 (− 8.82) nm; HRESIMS [M + Na]^+^ *m/z* 500.2768 (calcd for C_30_H_39_NO_4_Na 500.2771); ^1^H and ^13^C NMR data, found in Tables 1 and 2.

### Cytotoxicity Assay

With adriamycin as the positive control, compounds **1**, **4**–**9** were assessed for cytotoxicity against AGS, U251, and MDA-MB-231 by the CCK8 assay (Tables 3 and 4).

**Table 3:** The inhibition rates of compounds **1**, **4**‒**9** against LAG3/MHC II and LAG3/FGL1 at a concentration of 50 μM

	LAG3/MHC II (%)	LAG3/FGL1 (%)
**1**	82.41	83.94
**4**	55.72	24.90
**5**	85.32	79.11
**6**	− 6.96	2.66
**7**	82.65	92.70
**8**	78.93	92.49
**9**	77.42	58.36

**Table 4:** The IC_50_ values (μM) of compounds **1**, **5**, **7**, **8** against LAG3/MHC II and LAG3/FGL1

	LAG3/MHC II	LAG3/FGL1
**1**	4.74	11.78
**5**	3.18	
**7**	2.37	4.39
**8**	2.58	7.45

### LAG3/MHC II Binding Assay

Utilizing HTRF (Homogeneous Time-resolved Fluorescence) technology [34, 35], the inhibition activity of tested compounds interfering in the interaction between MHC II and LAG3 proteins was evaluated. The activity was measured using LAG3/MHC II binding assay kit commercially available from Cisbio. The tested compounds were dissolved with DMSO, then diluted with the diluent provided in the kit to give the tested solutions. 2 μL tested solutions, 4 μL Tag1-LAG3 protein, and 4 μL Tag2-MHC II protein were added in sterile 96-well dishes. After incubation for 15 min in room temperature, 5 μL anti-Tag1-Tb^3+^ and 5 μL anti-Tag2-XL665 (or 10 μL of pre-mixed anti-tag detection reagents) were added into each well. The concentration of DMSO in each well was kept below 0.5% and the final volume in each well was 20 μL in the moment. Then the 96-well dishes were kept incubating for 1 h to overnight at room temperature. Subsequently, remove the plate sealer and read specific emission signal at 665 nm on an HTRF compatible reader. The positive control was yielded by replacing tested solution with 2 μL diluent. The negative control consisted of 4 μL Tag2-MHC II protein, 5 μL anti-Tag1-Tb^3+^ and 5 μL anti-Tag2-XL665, as well as 6 μL diluent. In blank control, 5 μL anti-Tag1-Tb^3+^, 10 μL diluent, and 5 μL detection buffer were existed.

Using the following fluorescence intensity (F) measurements at 665 nm, such as the signals of positive control (F_p_), blank control (F_b_), and experimental groups (F_e_), the inhibition rate was calculated at each of the compound concentration levels. The inhibitory activity of compound against the interaction of LAG3 with MHC II was evaluated based on the formula as follow: Inhibition rate = (F_p_ − F_e_)/(F_p_ − F_b_) × 100%. IC_50_ values were calculated by linear regression of a dose-dependent inhibition rate curve.

### LAG3/FGL1 Binding Assay

The tested principles and methods were similar to those of LAG3/MHC II. Using LAG3/FGL1 binding assay kit from Cisbio, the inhibition activity of tested compounds on the binding between LAG3 and MHC II was evaluated. The ligand protein in this kit was Tag2-FGL1 protein, which was distinguished from Tag2-MHC II protein. Moreover, the compositions of tested groups, positive control, negative control, blank control were analogical with those of LAG3/MHC II kit. The experimental operations and statistical analysis were also identical with above-mentioned LAG3/MHC II kit.

## Electronic supplementary material

Below is the link to the electronic supplementary material.

Supplementary file1 (DOCX 12268 KB)
