# Supplementary material for: Cytochalasins from Xylaria sp. CFL5, an Endophytic Fungus of Cephalotaxus fortunei
Source: Nat Prod Bioprospect. 2020 Nov 4;11(1):87–98. doi: 10.1007/s13659-020-00279-5 (PMC7933320; doi:10.1007/s13659-020-00279-5)
Supplement: Supplementary file 1 — Supplementary file1 (DOCX 12268 KB) [file 13659_2020_279_MOESM1_ESM.docx]

**Supporting Information**

**Cytochalasins from *Xylaria sp*. CFL5, an endophytic fungus of *Cephalotaxus fortunei***

**Kai-Liang Ma**, **Shi-Hui Dong**, **Hang-Ying Li**, **Wen-Jun Wei**, **Yong-Qiang Tu**, **and Kun Gao^[[1]](#footnote-1)^***

*State Key Laboratory of Applied Organic Chemistry, College of Chemistry and Chemical Engineering, Lanzhou University, Lanzhou 730000, People’s Republic of China*

**Content**

[Figure S1. ^1^H NMR spectrum of compound **1** in CDCl_3_. 1](#_Toc51701430)

[Figure S2. ^13^C NMR spectrum of compound **1** in CDCl_3_. 1](#_Toc51701432)

[Figure S3. DEPT spectrum of compound **1** in CDCl_3_. 1](#_Toc51701433)

[Figure S4. ^1^H–^1^H COSY spectrum of compound **1** in CDCl_3_. 2](#_Toc51701434)

[Figure S5. HSQC spectrum of compound **1** in CDCl_3_. 3](#_Toc51701435)

[Figure S6. HMBC spectrum of compound **1** in CDCl_3_. 3](#_Toc51701436)

[Figure S7. NOESY spectrum of compound **1** in CDCl_3_. 4](#_Toc51701437)

[Figure S8. HRESIMS data of compound **1**. 4](#_Toc51701438)

[Figure S9. UV spectrum of compound **1**. 5](#_Toc51701439)

[Figure S10. IR spectrum of compound **1**. 5](#_Toc51701440)

[Figure S11. ^1^H NMR spectrum of compound **2** in CDCl_3_. 6](#_Toc51701441)

[Figure S12. ^13^C NMR spectrum of compound **2** in CDCl_3_. 6](#_Toc51701442)

[Figure S13. DEPT spectrum of compound **2** in CDCl_3_. 7](#_Toc51701443)

[Figure S14. ^1^H–^1^H COSY spectrum of compound **2** in CDCl_3_. 7](#_Toc51701444)

[Figure S15. HSQC spectrum of compound **2** in CDCl_3_. 8](#_Toc51701445)

[Figure S16. HMBC spectrum of compound **2** in CDCl_3_. 8](#_Toc51701446)

[Figure S17. NOESY spectrum of compound **2** in CDCl_3_. 9](#_Toc51701447)

[Figure S18. HRESIMS data of compound **2**. 9](#_Toc51701448)

[Figure S19. UV spectrum of compound **2**. 10](#_Toc51701449)

[Figure S20. IR spectrum of compound **2**. 10](#_Toc51701450)

[Figure S21. ^1^H NMR spectrum of compound **3** in CDCl_3_. 11](#_Toc51701451)

[Figure S22. ^13^C NMR spectrum of compound **3** in CDCl_3_. 11](#_Toc51701452)

[Figure S23. DEPT NMR spectrum of compound **3** in CDCl_3_. 12](#_Toc51701453)

[Figure S24. ^1^H–^1^H NMR spectrum of compound **3** in CDCl_3_. 12](#_Toc51701454)

[Figure S25. HSQC NMR spectrum of compound **3** in CDCl_3_. 13](#_Toc51701455)

[Figure S26. HMBC NMR spectrum of compound **3** in CDCl_3_. 13](#_Toc51701456)

[Figure S27. NOESY NMR spectrum of compound **3** in CDCl_3_. 14](#_Toc51701457)

[Figure S28. HRESIMS data of compound **3**. 14](#_Toc51701458)

[Figure S29. UV spectrum of compound **3**. 15](#_Toc51701459)

[Figure S30. IR spectrum of compound **3**. 15](#_Toc51701460)

[Figure S31. Six lowest energy conformers of compound **1** with (3*S*, 4*R*, 5*S*, 8*S*, 9*S*, 13*E*, 16*S*, 18*S*, 19*E*, 21*R*) configuration. 16](#_Toc51701461)

[Figure S32. Three lowest energy conformers of compound **2** with (3*S*, 4*R*, 5*S*, 8*S*, 9*S*, 13*E*, 16*R*, 18*S*, 19*E*, 21*R*) configuration. 16](#_Toc51701462)

[Figure S33. Three lowest energy conformers of compound **2** with (3*S*, 4*R*, 5*S*, 8*S*, 9*S*, 13*E*, 16*S*, 18*R*, 19*E*, 21*R*) configuration. 16](#_Toc51701463)

[Figure S34. Five lowest energy conformers of compound **3** with (3*S*, 4*R*, 5*S*, 8*S*, 9*S*, 13*E*, 16*S*, 17*R*, 18*S*, 19*E*, 21*R*) configuration. 17](#_Toc51701464)

[Table S1. Calculated optical rotation for compound **2**. 17](#_Toc51701465)

[ORD Calculation Method 17](#_Toc51701466)

[ECD Calculation Method 18](#_Toc51701467)

## Figure S1. ^1^H NMR spectrum of compound **1** in CDCl_3_.

## Figure S2. ^13^C NMR spectrum of compound **1** in CDCl_3_.

## Figure S3. DEPT spectrum of compound **1** in CDCl_3_.

## Figure S4. ^1^H–^1^H COSY spectrum of compound **1** in CDCl_3_.

## Figure S5. HSQC spectrum of compound **1** in CDCl_3_.

## Figure S6. HMBC spectrum of compound **1** in CDCl_3_.

## Figure S7. NOESY spectrum of compound **1** in CDCl_3_.

## Figure S8. HRESIMS data of compound **1**.

## Figure S9. UV spectrum of compound **1**.


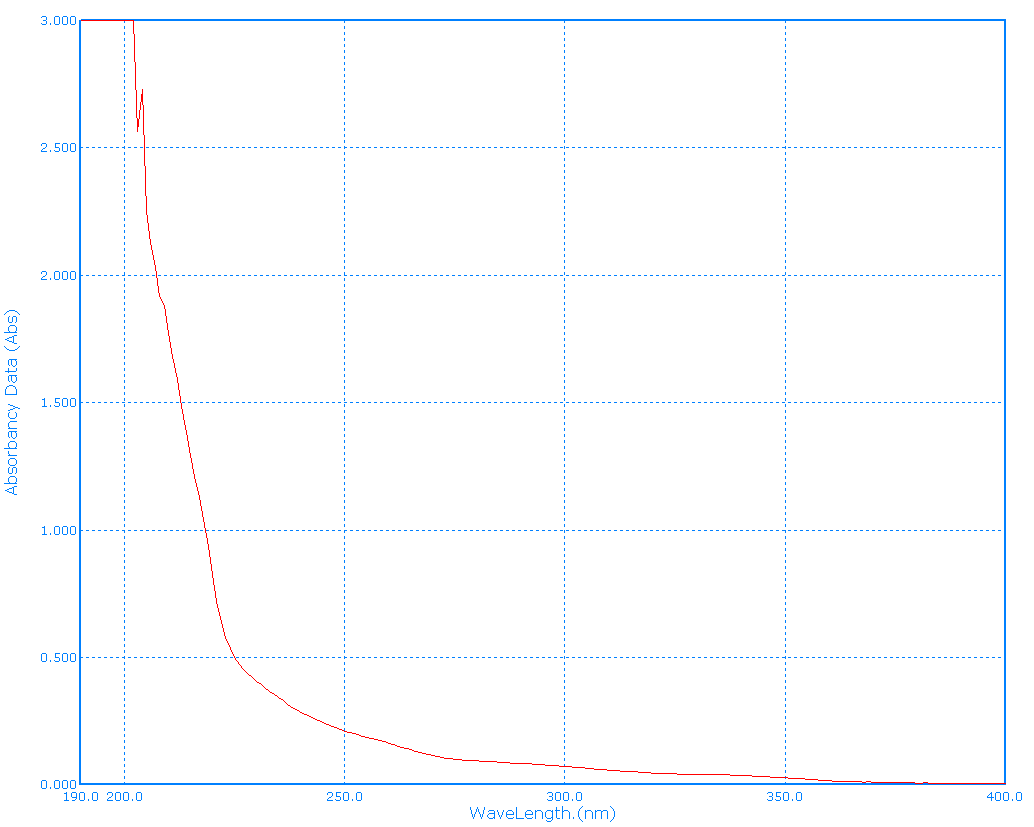


## Figure S10. IR spectrum of compound **1**.


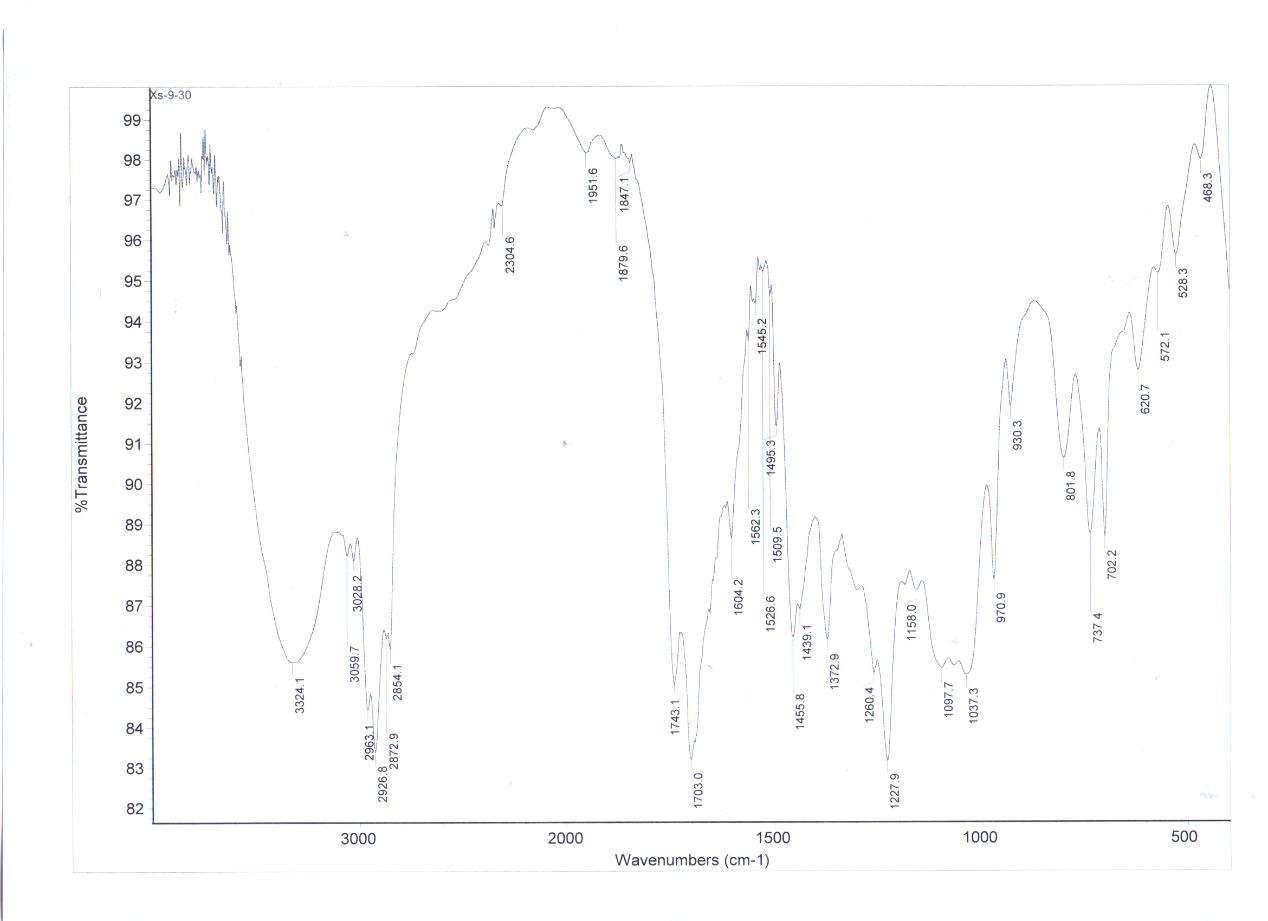


## Figure S11. ^1^H NMR spectrum of compound **2** in CDCl_3_.

## Figure S12. ^13^C NMR spectrum of compound **2** in CDCl_3_.

## Figure S13. DEPT spectrum of compound **2** in CDCl_3_.

## Figure S14. ^1^H–^1^H COSY spectrum of compound **2** in CDCl_3_.

## Figure S15. HSQC spectrum of compound **2** in CDCl_3_.

## Figure S16. HMBC spectrum of compound **2** in CDCl_3_.

## Figure S17. NOESY spectrum of compound **2** in CDCl_3_.

## Figure S18. HRESIMS data of compound **2**.

## Figure S19. UV spectrum of compound **2**.


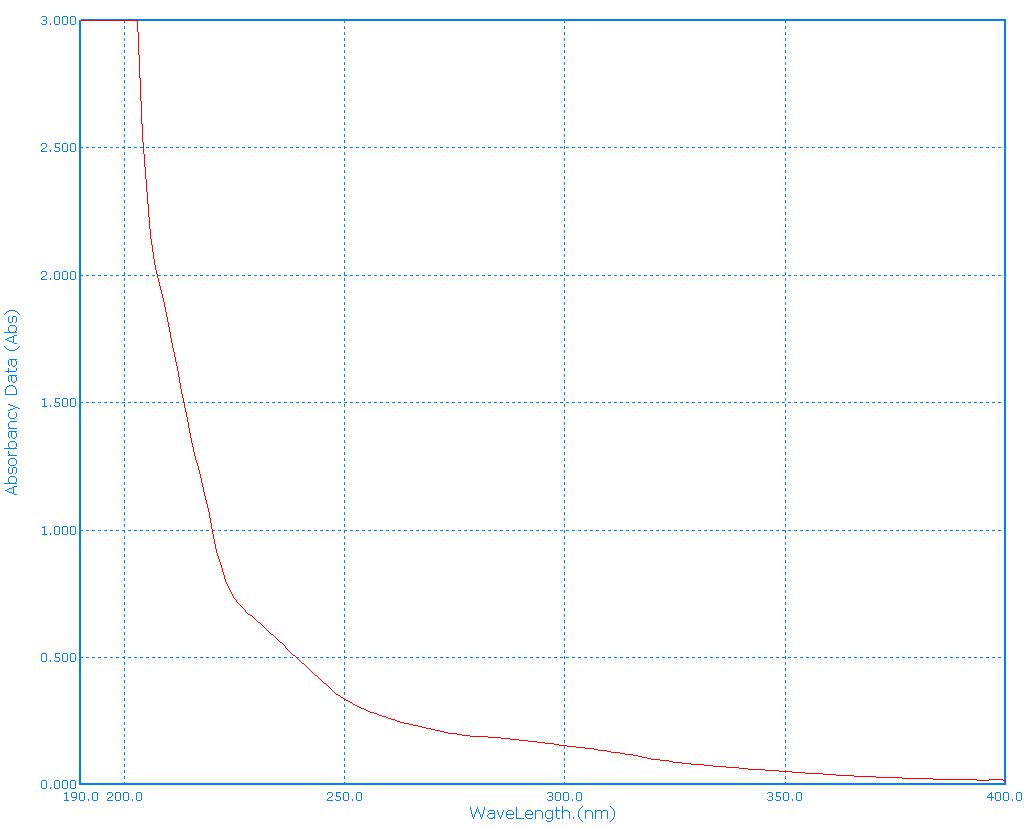


## Figure S20. IR spectrum of compound **2**.


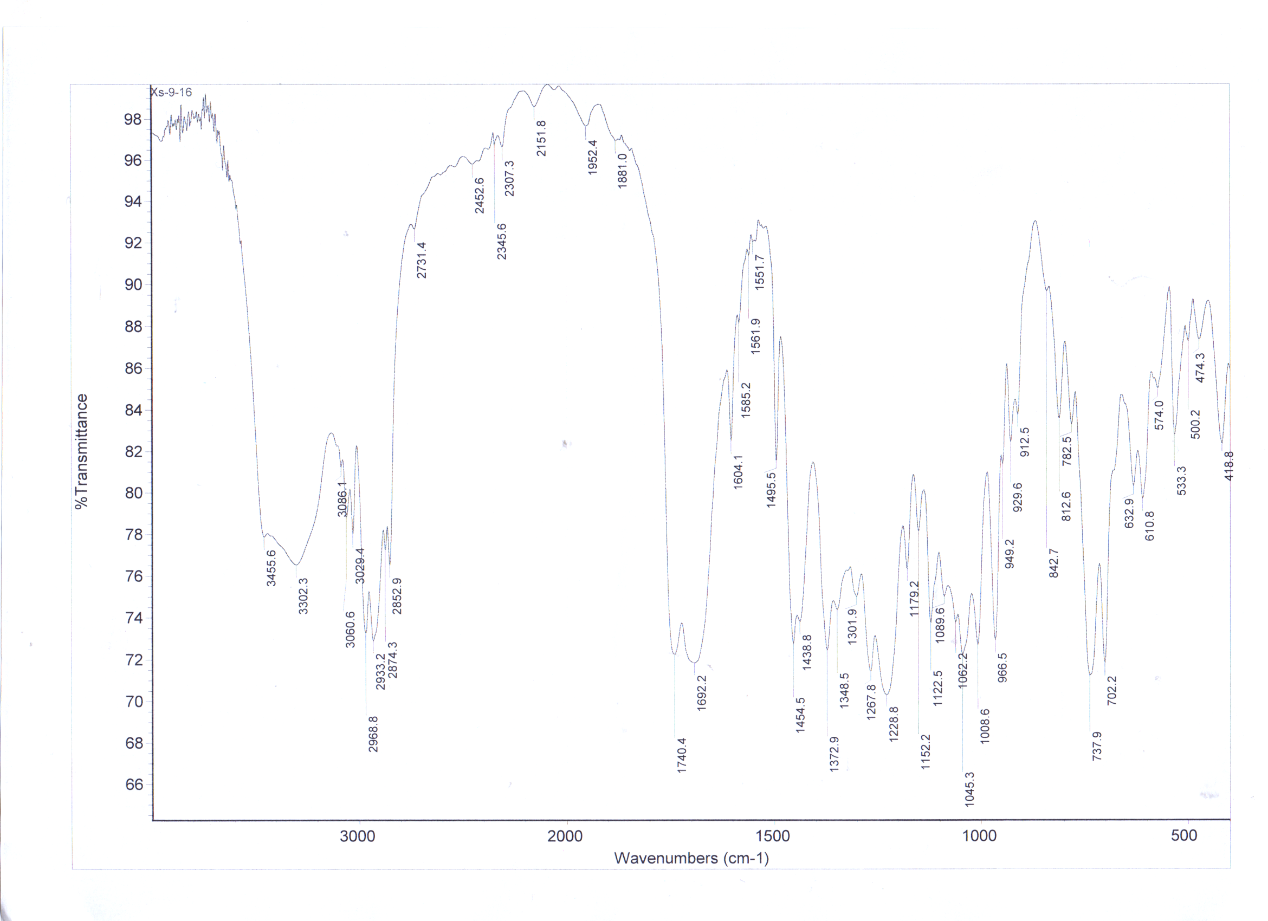


## Figure S21. ^1^H NMR spectrum of compound **3** in CDCl_3_.

## Figure S22. ^13^C NMR spectrum of compound **3** in CDCl_3_.


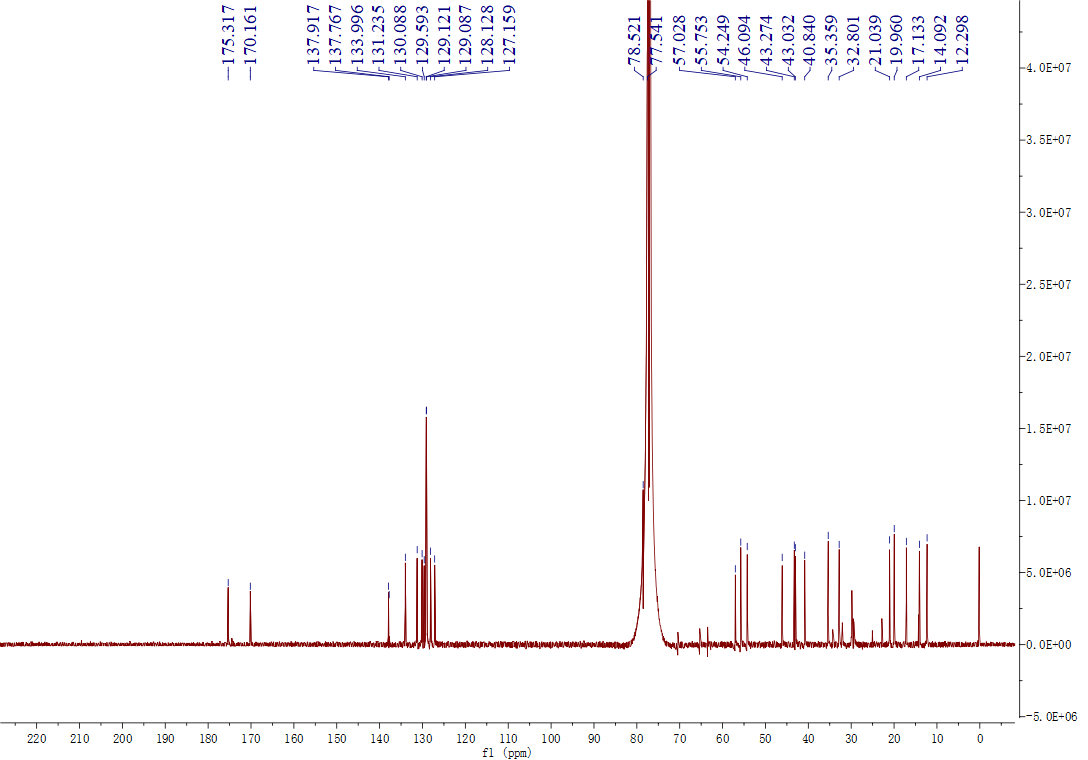


## Figure S23. DEPT NMR spectrum of compound **3** in CDCl_3_.

## Figure S24. ^1^H–^1^H NMR spectrum of compound **3** in CDCl_3_.

## Figure S25. HSQC NMR spectrum of compound **3** in CDCl_3_.


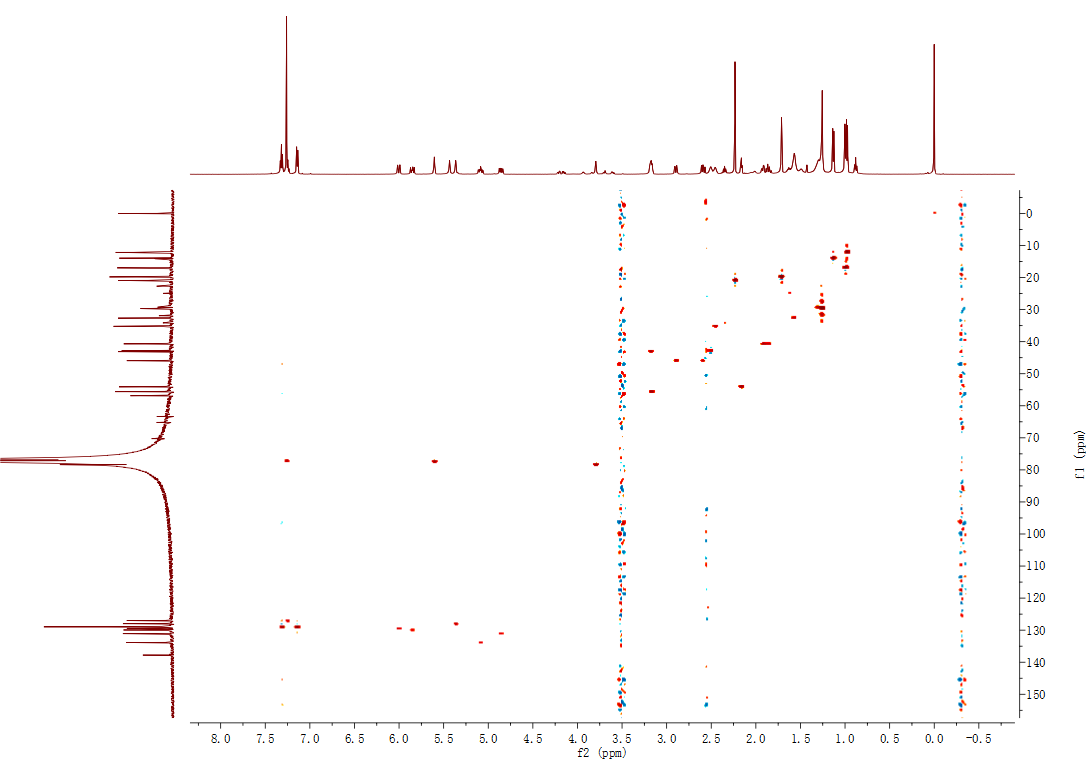


## Figure S26. HMBC NMR spectrum of compound **3** in CDCl_3_.


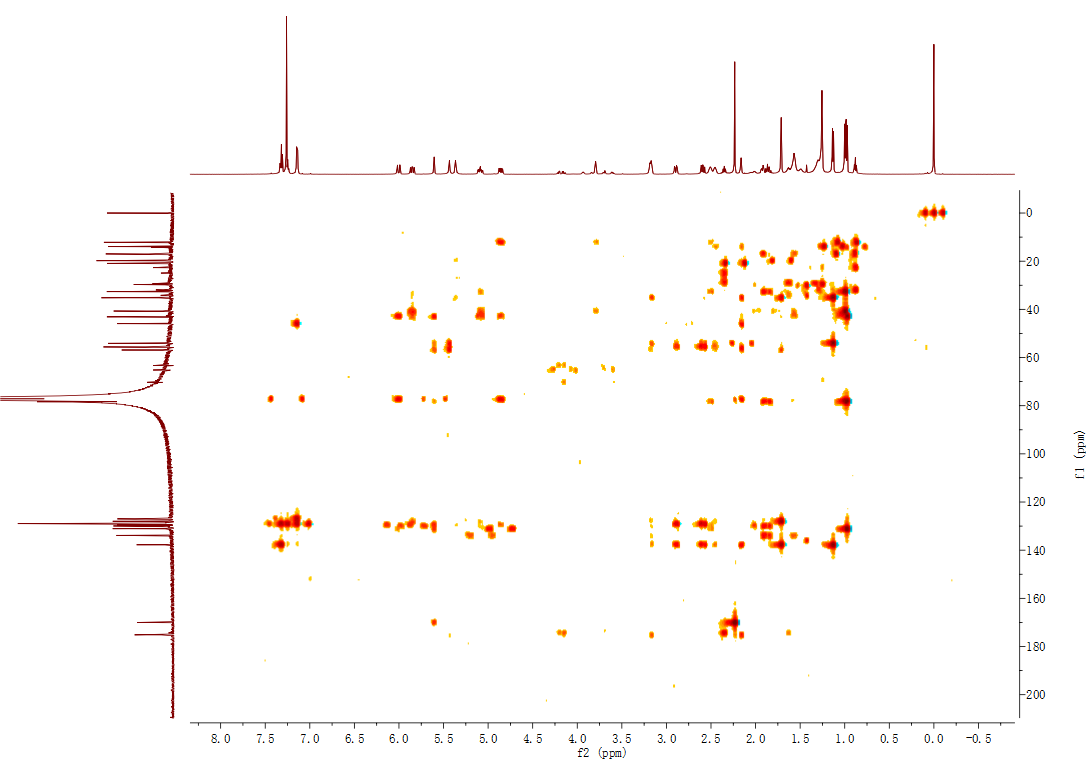


## Figure S27. NOESY NMR spectrum of compound **3** in CDCl_3_.

## Figure S28. HRESIMS data of compound **3**.

## Figure S29. UV spectrum of compound **3**.


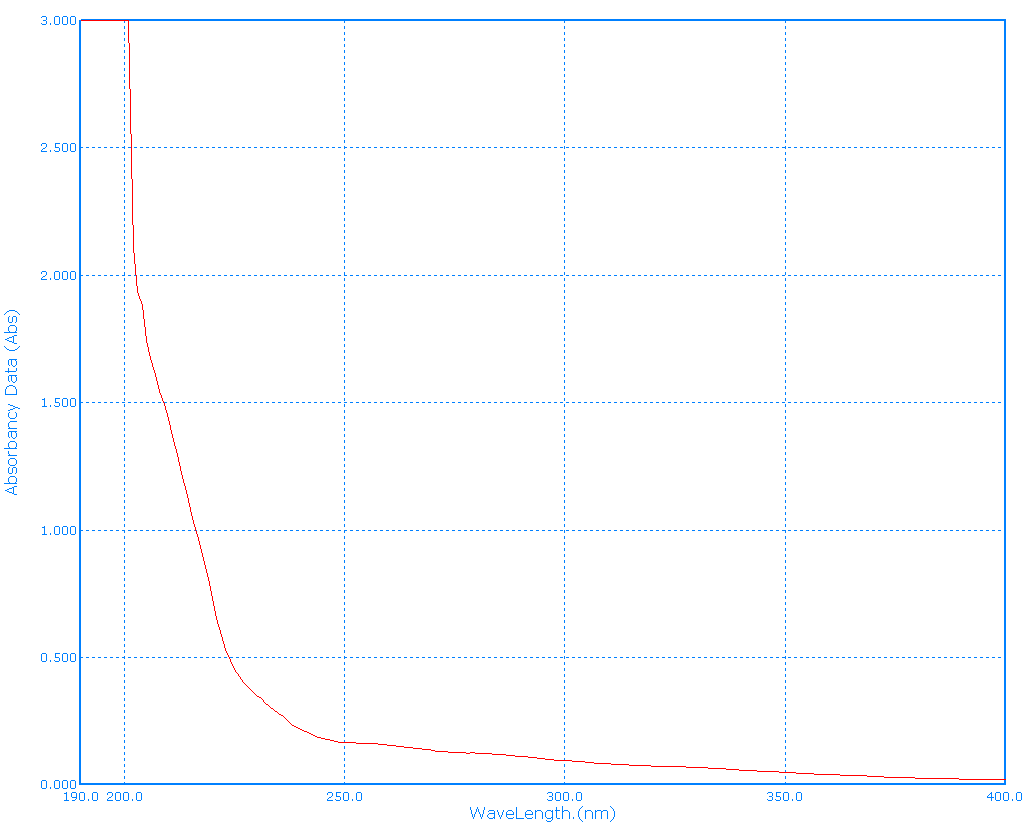


## Figure S30. IR spectrum of compound **3**.


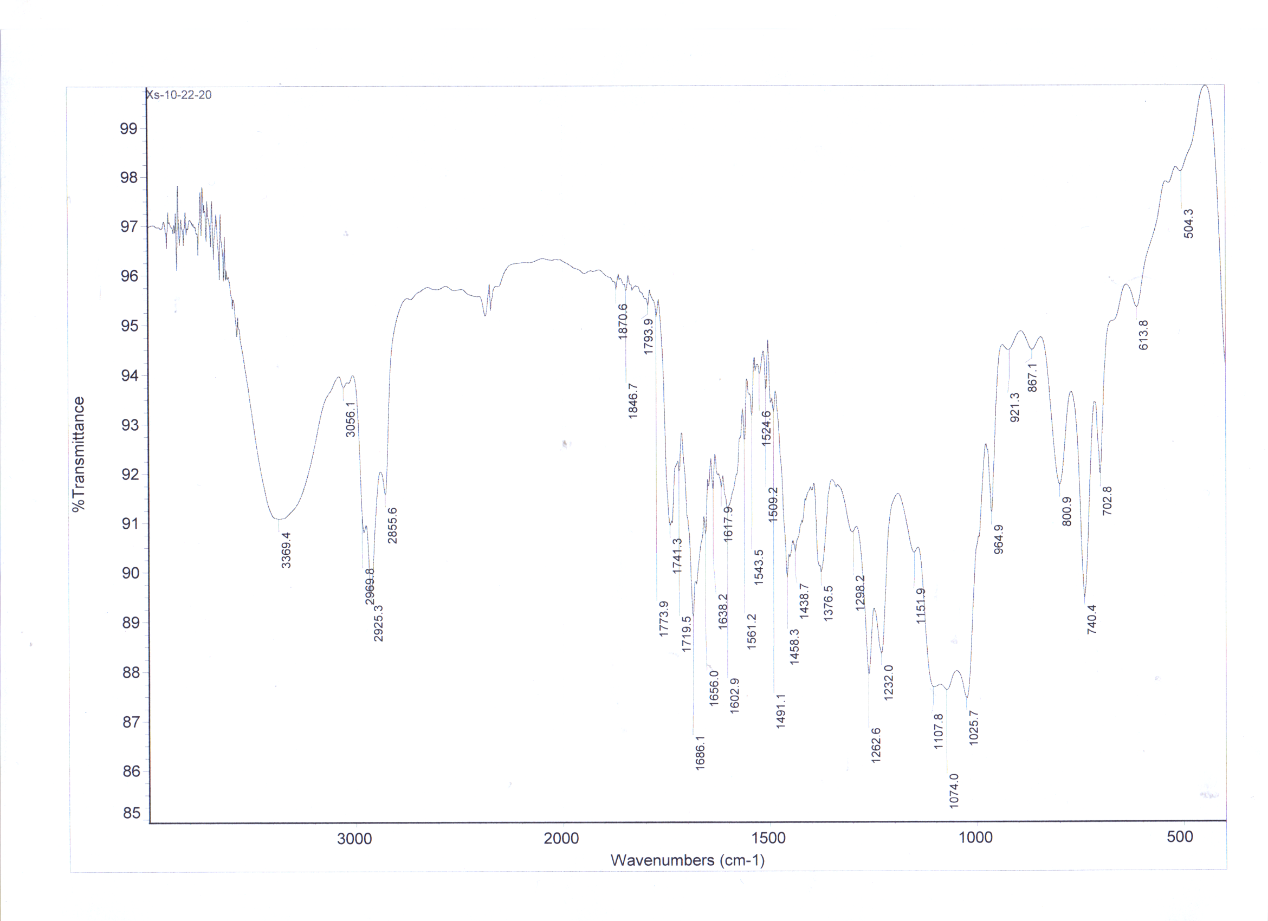


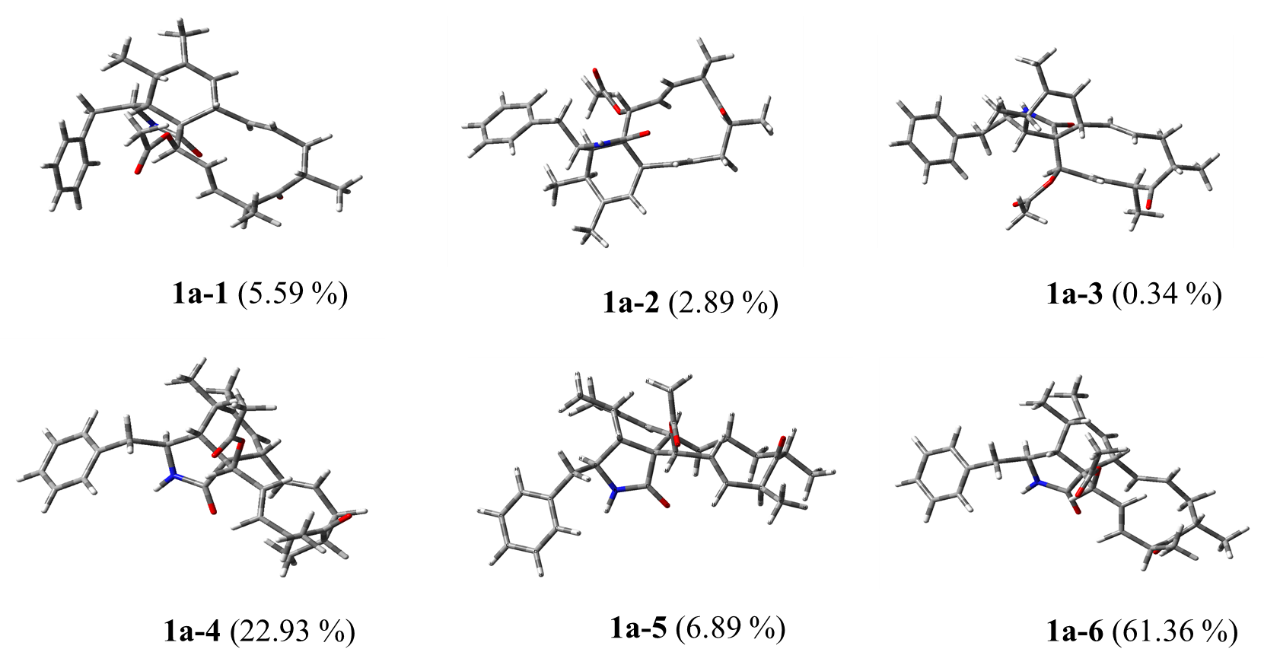


## Figure S31. Six lowest energy conformers of compound **1** with (3*S*, 4*R*, 5*S*, 8*S*, 9*S*, 13*E*, 16*S*, 18*S*, 19*E*, 21*R*) configuration.


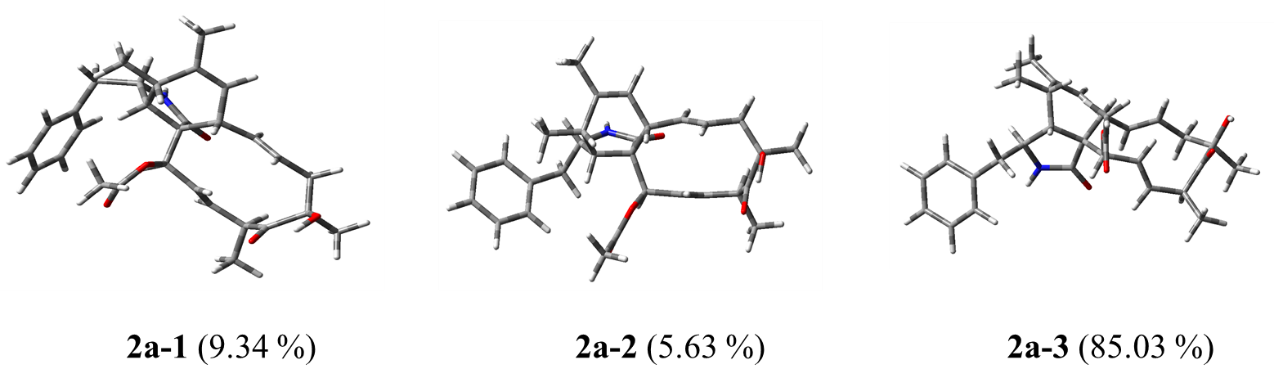


## Figure S32. Three lowest energy conformers of compound **2** with (3*S*, 4*R*, 5*S*, 8*S*, 9*S*, 13*E*, 16*R*, 18*S*, 19*E*, 21*R*) configuration.


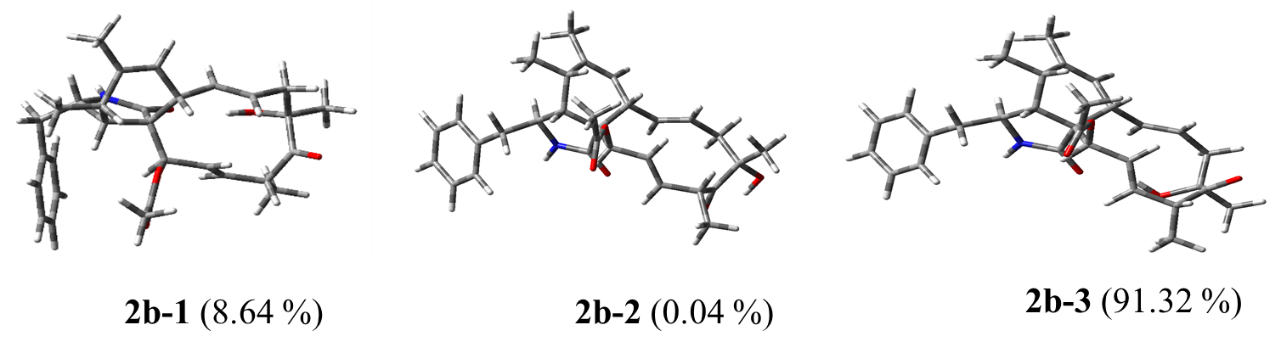


## Figure S33. Three lowest energy conformers of compound **2** with (3*S*, 4*R*, 5*S*, 8*S*, 9*S*, 13*E*, 16*S*, 18*R*, 19*E*, 21*R*) configuration.


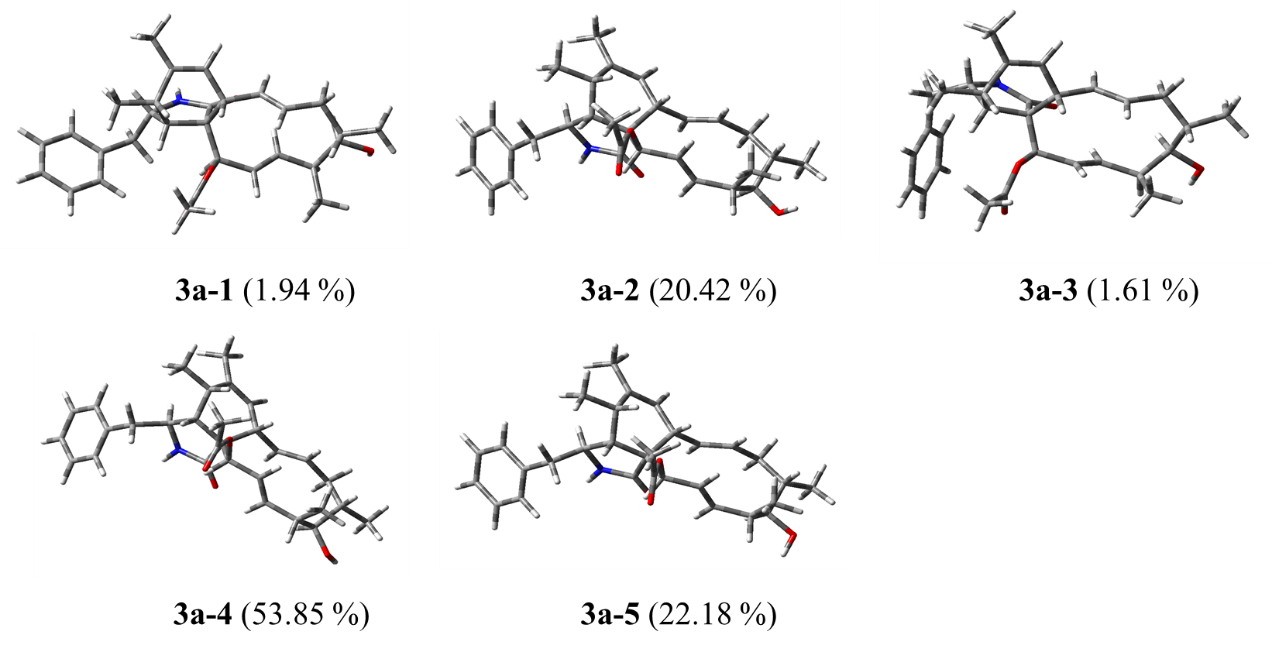


## Figure S34. Five lowest energy conformers of compound **3** with (3*S*, 4*R*, 5*S*, 8*S*, 9*S*, 13*E*, 16*S*, 17*R*, 18*S*, 19*E*, 21*R*) configuration.

## Table S1. Calculated optical rotation for compound **2**.

| compound | conformers | percentage (%) | specific rotation | fitting result |
| --- | --- | --- | --- | --- |
| **2a** | **2a-1** | 9.34 | 74.13 | -44.00 |
|  | **2a-2** | 5.63 | 158.07 |  |
|  | **2a-3** | 85.03 | -70.35 |  |
| **2b** | **2b-1** | 8.64 | 169.87 | 9.52 |
|  | **2b-2** | 0.04 | -201.75 |  |
|  | **2b-3** | 91.32 | -5.56 |  |

**ORD Calculation**

## ORD Calculation Method

Conformation search for compounds were performed by Spartan’s 14 using Merk Molecular Force Field (MMFF) level. The low energy conformations of compounds were submitted to the density functional theory (DFT) optimization at the level of b3lyp/6-31g(d,p), using the pcm solvation model with the dielectric constant representing Methanol. The optimized structures were subject to the frequency calculations at b3lyp/6-31g(d,p) level to confirm the true energy minimal located and generate the thermodynamic data. The optimized structures were further submitted to the Time-dependent density functional theory (TDDFT) calculations at b3lyp/6-31g(d,p). The simulated ORD value of the conformers were averaged according to the Boltzmann distribution theory and their relative Gibbs free energy.

**ECD Calculation**

## ECD Calculation Method

Conformation search for compounds were performed by Spartan’s 14 using Merk Molecular Force Field (MMFF) level. The low energy conformations of compounds were submitted to the density functional theory (DFT) optimization at the level of b3lyp/6-31g(d,p), using the cpcm solvation model with the dielectric constant representing Methanol. The optimized structures were subject to the frequency calculations at b3lyp/6-31g(d,p) level to confirm the true energy minimal located and generate the thermodynamic data. The optimized structures were further submitted to the Time-dependent density functional theory (TDDFT) calculations at b3lyp/6-31g(d,p). Rotatory strengths for a total 100 excited states were calculated. The simulated ECD spectra of the conformers were averaged according to the Boltzmann distribution theory and their relative Gibbs free energy, which was generated using the SpecDis 1.53 and GraphPad Prism 5 from dipole-length rotational strengths by applying Gaussian band shapes with = 0.3 ev.

1. * Corresponding author,

   E-mail: [npchem@lzu.edu.cn](mailto:npchem@lzu.edu.cn) (K. Gao) [↑](#footnote-ref-1)
